# Monomeric Zn(II) and 3D polymeric Ag(I) complexes with a cationic *s*-triazine hydrazone-based ligand; synthesis, structure, coordination behavior and biological studies

**DOI:** 10.1038/s41598-026-57114-z

**Published:** 2026-08-11

**Authors:** Eman M. Fathalla, Amal Yousri, Ayman El-Faham, Saied M. Soliman, Assem Barakat, Roland C. Fischer, Morsy A.M. Abu-Youssef, Ahmed M. A. Badr, Sara M. Khattab

**Affiliations:** 1 Department of Chemistry, Faculty of Science, Alexandria University, P.O. Box 426, Ibrahimia, Alexandria, 21321 Egypt; 2 Department of Basic Medical Sciences, College of Medicine, Dar Al Uloom University, P. O. Box 45142, 11512 Riyadh, Saudi Arabia; 3 Department of Chemistry, College of Science, King Saud University, P.O. Box 2455, 11451 Riyadh, Saudi Arabia; 4 Institute of Inorganic Chemistry, Graz University of Technology, Stremayrgasse 9/V, A-8010 Graz, Austria

**Keywords:** Cationic ligand, Structure diversity, 3D polymeric Ag(I) complex, Hirshfeld surface analysis, Cytotoxicity, Antimicrobial, Biochemistry, Cancer, Chemistry, Drug discovery

## Abstract

The novel (*E*)-6-(2-(1-(pyridin-4-yl)ethylidene)hydrazineyl)-1,3,5-triazine-2,4-diamine (**L**) was used to synthesize the monomeric [Zn(HL)Cl_3_] (**1**) and the 3D polymeric [Ag_4_(HL)_2_(NO_3_)_6_]_n_ (**2**) complexes. Their structures were verified through single-crystal X-ray diffraction. In both complexes, **L** is found protonated leading to the cationic coordinating ligand (**HL**^+^). **1** exhibits a slightly distorted tetrahedral geometry (τ_4_ = 0.93 and τ_4_′=0.92) while **2** contains four crystallographically non-equivalent Ag(I) ions. Ag1 has a square pyramidal geometry (τ_5_ = 0.23), Ag2 exhibits a distorted tetrahedron (τ_4_ = 0.55 and τ_4_′=0.54), Ag3 and Ag4 possess a distorted trigonal planar geometry. Hirshfeld surface analysis reveals that hydrogen-based interactions primarily dictate the molecular packing of complexes **1** and **2** (81.3% and 69.2%, respectively). Theoretical NBO analysis was conducted to quantify the flux of electrons from the donor to the acceptor and evaluate the orbital-orbital interactions of the coordinate bonds in both complexes. Regarding to cytotoxicity, **2** achieved impressive results with IC_50_ values of 9.96 ± 0.35 and 15.84 ± 0.46 *µ*M against HepG-2 and MCF-7 cell lines, respectively, surpassing *cis*-platin by six and four times, respectively. Additionally, **2** exhibits broad-spectrum antimicrobial activity, achieving the most significant activity against *B. subtilis*, with a diameter of inhibition zone of 27 mm and MIC value of 3.71 *µ*M, comparable to that of gentamicin as a positive control.

**Supplementary Information:**

The online version contains supplementary material available at 10.1038/s41598-026-57114-z.

## Introduction

The fundamental aim of medicinal chemistry is to obtain compounds of high bioactivity and bioavailability. It is well documented that nitrogen-containing heterocycles especially *s*-triazine derivatives are of great interest for diverse biological activities^1,2^. Comparisons show that medications based on *s-*triazine are more potent than their non-triazine counterparts^3^. In consequence, dendrimers based on melamine (2,4,6-triamino-*s*-triazine) are widely used in cancer therapies and other biological treatments^4^. Moreover, in the realm of neuroleptics, *s*-triazines have demonstrated the strongest anti-methamephetamine effects while being less toxic than chlorpromazine^5^. Furthermore, hydrazone-Schiff bases derivatives have a significant function in drug design and development because of their diverse structures, ease of synthesis, and ability to modify molecular properties^6–8^. The value of these derivatives in drug development primarily lies in their potential to enhance therapeutic strategies *via* targeted and optimized drug design, along with their diverse applications^9–11^. Beyond their medicinal applications, hydrazone-Schiff base derivatives also serve as effective metal ion chelators, contributing to chelation therapy for diseases associated with metal overload^12^.

On the other hand, cationic ligands, which have a positive charge on or near the coordinating atom, are uncommon in coordination chemistry. This rarity is due to the destabilizing repulsion between the cationic ligand and the typically positively charged metal ion, making such ligands less documented in traditional coordination and organometallic chemistry. Some documented examples are α-cationic phosphines^13^ and nitrenium cations^14^. In recent years, a few studies focusing on cationic *N*-heterocyclic carbenes (NHCs)^15^ and other cationic nitrogen donor ligands^16–19^ have been reported. These ligands can be protonated at one of their N-sites which critically affects the donor atoms participating in the coordination with metal ion^16^. In literature, studies have shown that protonated ligands typically form upon acid addition, as observed in Zn(II) and Co(II) complexes with imidazole-1,2,4-triazole ligand^16^, as well as in Ni(II)^17,19^ and Ru(II)^18^ complexes with 2,4,6-tri-2-pyridyl-1,3,5-triazine ligand.

Furthermore, complexes with coinage metals, such as those with silver, often demonstrate increased biological activity when coordinated with hydrazone ligands, including selective anticancer and strong antimicrobial effects towards resistant pathogens^20^. Besides, in the silver complexes, the presence of ligands and multiple silver ions enhances the biological activity^21^. Recent studies suggest that silver complexes are more effective anticancer agents for various cancers, such as ovarian, breast, lung, and colorectal cancer than *cis*-platin^22,23^. Due to the d^10^ electronic configuration of Ag(I) and Zn(II), they can adopt a variety of geometries^24,25^, with coordination number and shape influenced solely by ligand size and charge^26^. Biological effectiveness of several Zn(II) complexes of hydrazone-Schiff bases^27^, *s*-triazine and pyridine-based ligands^28^ have been investigated.

**Scheme 1:**
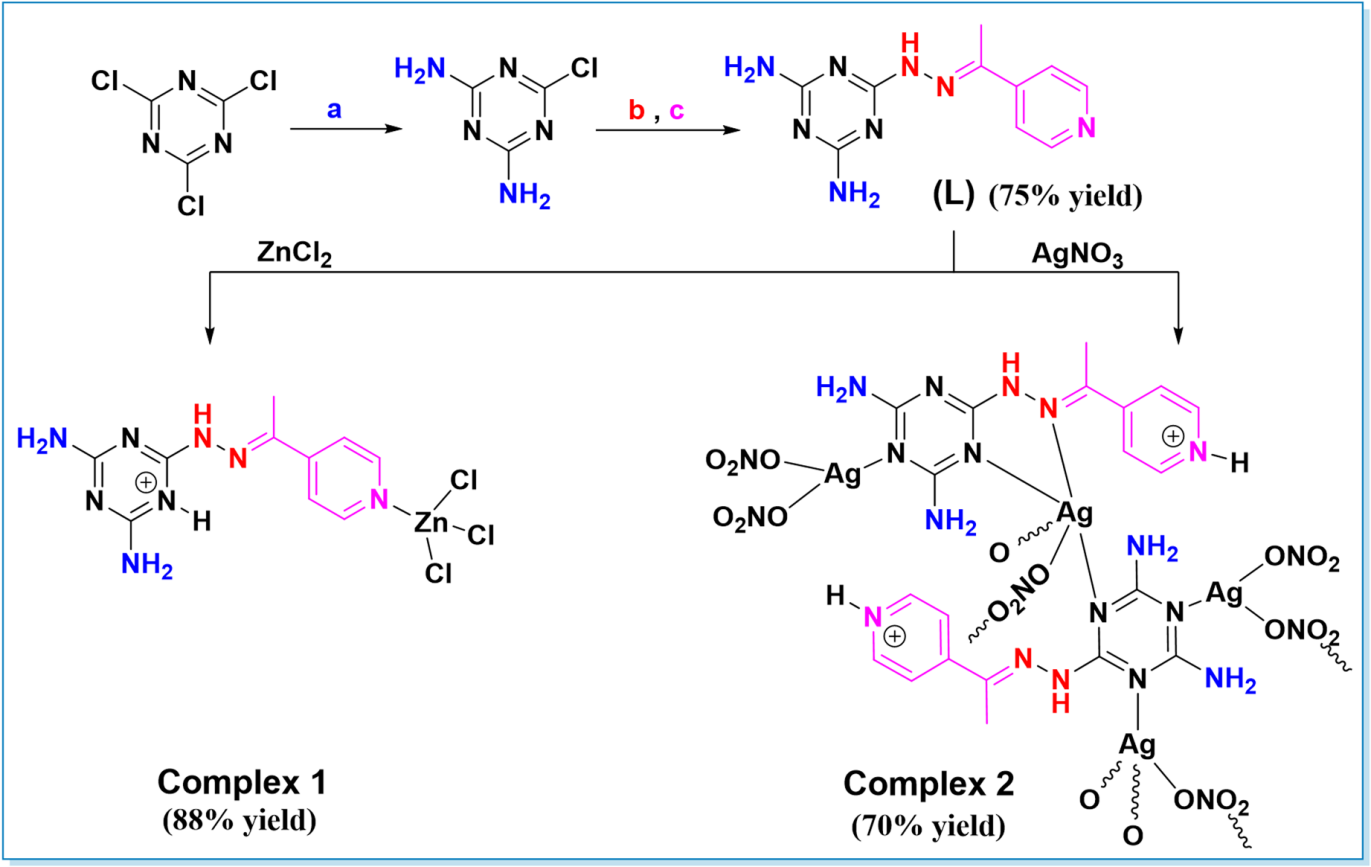
Reactions pathways illustrating the synthesis of **L** and its two complexes; (**a**) NH_4_OH, acetone/ water, 0–40 °C, 1 h; (**b**) NH_2_-NH_2_.H_2_O, water, 85 °C, overnight; (**c**) 4-acetylpyridine, methanol, rt, overnight.

In the light of the rich structural diversity and remarkable biological activities of Ag(I) and Zn(II) complexes in addition to hydrazone Schiff bases, *s*-triazine, and pyridine compounds, this study presents an approach to synthesizing a novel *s*-triazine hydrazone-based ligand (**L**, Scheme 1). The ligand (**L**) was then utilized to construct novel Ag(I) and Zn(II) complexes, whose structural and electronic features were investigated using single crystal X-ray diffraction, Hirshfeld and spectroscopic analyses combined with NBO calculations. Finally, their anticancer and antibacterial activities were evaluated.

## Results and discussion

### Synthesis and characterization

A novel organic ligand, (*E*)-6-(2-(1-(pyridin-4-yl)ethylidene)hydrazineyl)-1,3,5-triazine-2,4-diamine (**L**), was synthesized in three steps, using cyanuric chloride as the raw substance (Scheme 1). The first step involves substituting two chlorine atoms from the cyanuric chloride with two amine groups using ammonium hydroxide as a reagent, at a temperature ranging from 0 to 40 °C. In the second step, the third chlorine atom is replaced with hydrazine, which is achieved by refluxing overnight at 85 °C. The third step involves the preparation of the Schiff base by reacting the previous compound with 4-acetylpyridine, using methanol as the solvent with stirring overnight at room temperature. At the end, the target organic compound (**L**) was obtained with high purity and a yield of 75%. Following that, this synthesized organic compound was used to prepare two metal complexes (Scheme 1) *via* the self-assembly technique. The two complexes were prepared as crystals and then analyzed using single crystal XRD, NMR, FT-IR, and elemental analyses. In the two complexes, the ligand is found to be protonated at one of its N-atoms leading to the cationic formula (**HL**^+^). Notably, the protonation of the ligand occurred without the addition of any external acid, utilizing an aqueous ethanolic solution during synthesis. This finding correlates with literature^29^ indicating that the coordination of a d^10^ metal ion such as Zn²⁺ to water clusters enhances their acidity, leading to unimolecular proton release.

### FT-IR spectra

Notable shifts are observed in the FT-IR spectrum of both the complexes when compared to the free ligand **L** (Figs. S1–S3). For **L**, the N-H stretching vibrations of NH_2_ is represented by two peaks at 3364 and 3196 cm^−130^. These relatively sharp peaks appeared at 3432 and 3346 cm^−1^ in case of **1**, while are observed at 3318 and 3216 cm^−1^ in **2**. In addition, the C=N stretching vibrations are observed at 1584 and 1540 cm^−1^ for **L**. These peaks appeared at 1586 and 1541 cm^−1^ in case of **1**, indicating that the azomethine group does not participate in the coordination^31^. In contrast, the C=N stretching modes in **2** are shifted to higher wavenumbers of 1628 and 1593 cm^−1^, supporting the involvement of the azomethine group in the coordination^32^. These findings are in agreement with the proposed X-ray structures. The C=C stretching vibrations are present at 1478 cm^−1^ for **L** and at 1532 cm^−1^ for **2**, whereas in **1**, this peak splits into two bands at 1491 and 1463 cm^−1^. Furthermore, **2** shows a sharp peak at 1377 cm^−1^, indicating the presence of NO_3_^−^ group^33^. Additionally, a new peak at 3517 cm^−1^ arises from the O-H stretching vibration of a half ethanol molecule in **1**.

### NMR spectra

The NMR analysis confirmed the structure of the synthesized ligand (**L**) and the two complexes (Figs. S4–S9). ^1^H NMR spectra of **L**, **1** and **2** were measured in DMSO-*d*_*6*_ and DMSO-*d*_*6*_/D_2_O. Chemical shift changes were observed when comparing the ligand’s spectrum with those of the two complexes, confirming the coordination with the metal ion^34,35^. **L** displays a signal at δ 9.57 ppm, which is attributed to the NH proton. Notably, this signal shifts to δ 9.97 and 10.74 ppm in complexes **1** and **2**, respectively. Additionally, the signal found at δ 6.37 ppm for **L** is attributed to –NH_2_ protons. In the spectrum of **2**, this signal shifts to δ 7.22 ppm, whereas in **1** it appears at δ 6.56 ppm. The peaks related to the NH and NH_2_ proton of the ligand and its two complexes are exchangeable with D_2_O as shown in Figs. S4–S6. For **L**, the signals between δ 8.54 and 7.66 ppm are assigned to the CH protons of the pyridine moiety. These signals shifted to δ 8.55–7.77 ppm in **1**, while they appeared in the range of 8.18–7.60 ppm in **2**. Moreover, the coordination of Zn(II) and Ag(I) ions with **L** was further evidenced by the observed shifts in their ^13^C NMR spectra compared to the free ligand **L**^36,37^ (Figs. S7–S9). The triazine carbons in **L**, observed at δ 167.9 and 165.9 ppm, exhibit slight shifts to δ 167.5 and 165.4 ppm in **1** and substantial shifts to δ 166.1 and 163.6 ppm in **2**. These shifts are consistent with the involvement of the triazine moiety in coordination with the Ag(I) ion in **2**. In **L**, the C=N (Schiff base residue) resonates at δ 150.2 ppm, remaining nearly unchanged at δ 150.3 ppm in **1**, but shifting downfield to δ 151.5 ppm (Δδ = 1.3 ppm) in **2**. This downfield displacement indicates coordination of the azomethine nitrogen^38,39^ to the Ag(I) center in **2**. Additionally, the pyridine carbons at δ 146.4, 144.1, and 120.7 ppm in **L** are shifted to δ 147.4, 121.7, and 100.0 ppm in **1** and δ 148.2, 122.8, and 100.0 ppm in **2**. The carbon of the methyl group at δ 13.3 ppm in **L** exhibits downfield at δ 13.5 and 16.5 ppm in **1** and **2**, respectively.

The observed changes in the ¹H NMR and ^13^C NMR chemical shifts can be attributed to two possible factors: (i) metal-ligand coordination (ii) protonation of the organic ligand. Both factors affect the ligand’s electronic environment leading to some variations in the NMR chemical shifts. It is well established in the literature that Ag(I) complexes with N-heterocyclic ligands typically exhibit minimal changes in ¹H NMR chemical shift (≤ 0.06 ppm) upon coordination^35,40^, a behavior attributed to the relatively weak Ag-N and Ag-O contacts^41^. In contrast, significantly larger shifts were observed in the present study. Therefore, the presence of the protonated ligand in solution is likely a main contributing factor to these unexpectedly significant chemical shift variations. The detailed structural analysis of the two complexes, along with the existence of the ligand in a protonated cationic form (**HL**^+^), was confirmed through single crystal X- ray studies.

### X-ray structure description

#### Structure of [Zn(HL)Cl_3_] complex (**1**)

**Fig. 1:**
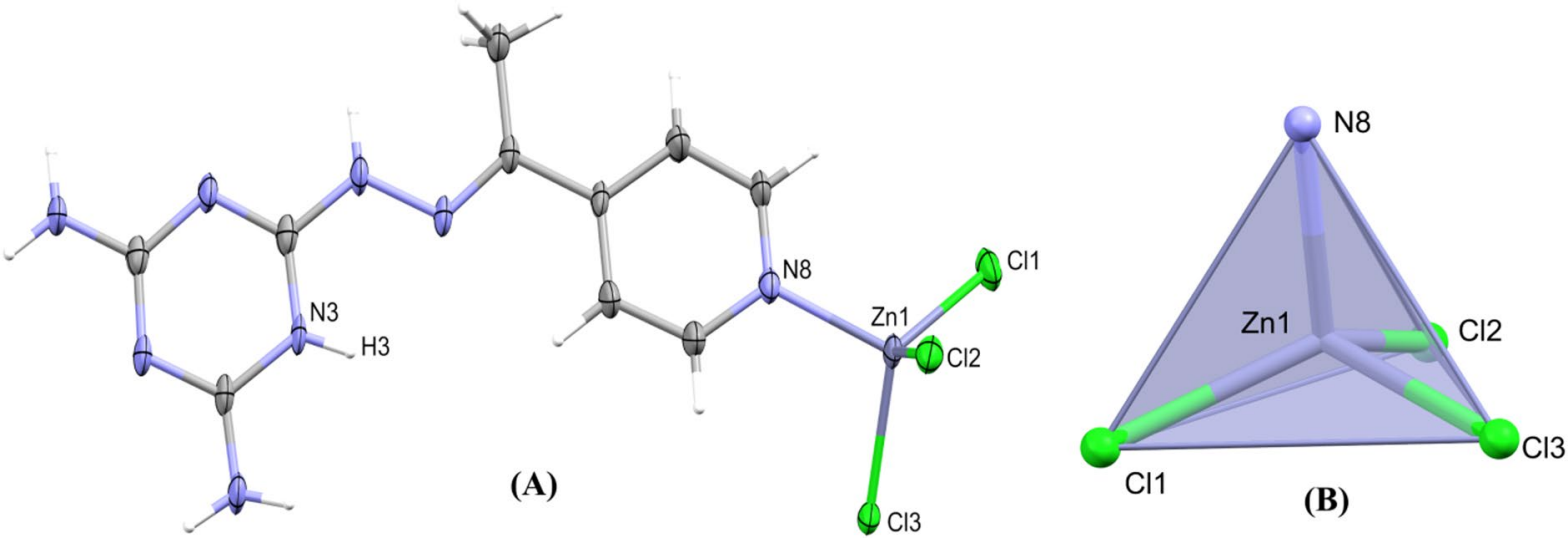
X-ray structure showing atom numbering (**A**) and the distorted tetrahedral configuration (**B**) of [Zn(HL)Cl_3_] complex (**1**). ORTEP diagram, illustrating thermal ellipsoids at 50% probability level, is shown in Fig. S10.

Single-crystal XRD technique revealed that **1** (CCDC 2518341) crystallizes in the monoclinic crystal system with the *C2/c* space group (Table S1). The asymmetric formula of this complex is represented as [Zn(HL)Cl_3_].0.5C_2_H_5_OH. Since the half ethanol molecule was heavily disordered, it was removed using the PLATON SQUEEZE procedure^42^. As a result, the formula [Zn(HL)Cl_3_] is used to describe the crystal data of complex **1** (Fig. 1). As illustrated in this figure, the ligand (**L**) is found protonated at N3 atom of the *s-*triazine moiety leading to the cationic formula (**HL**^**+**^). As a result, the pyridyl N-atom of **HL**^**+**^ is the donor atom which is found coordinated with Zn(II) center. In addition, three Cl^−^ ions complete the coordination sphere resulting in a neutral tetra-coordinated [Zn(HL)Cl_3_] formula. Table 1 provides a list of the specific bond distances and angles.

**Fig. 2:**
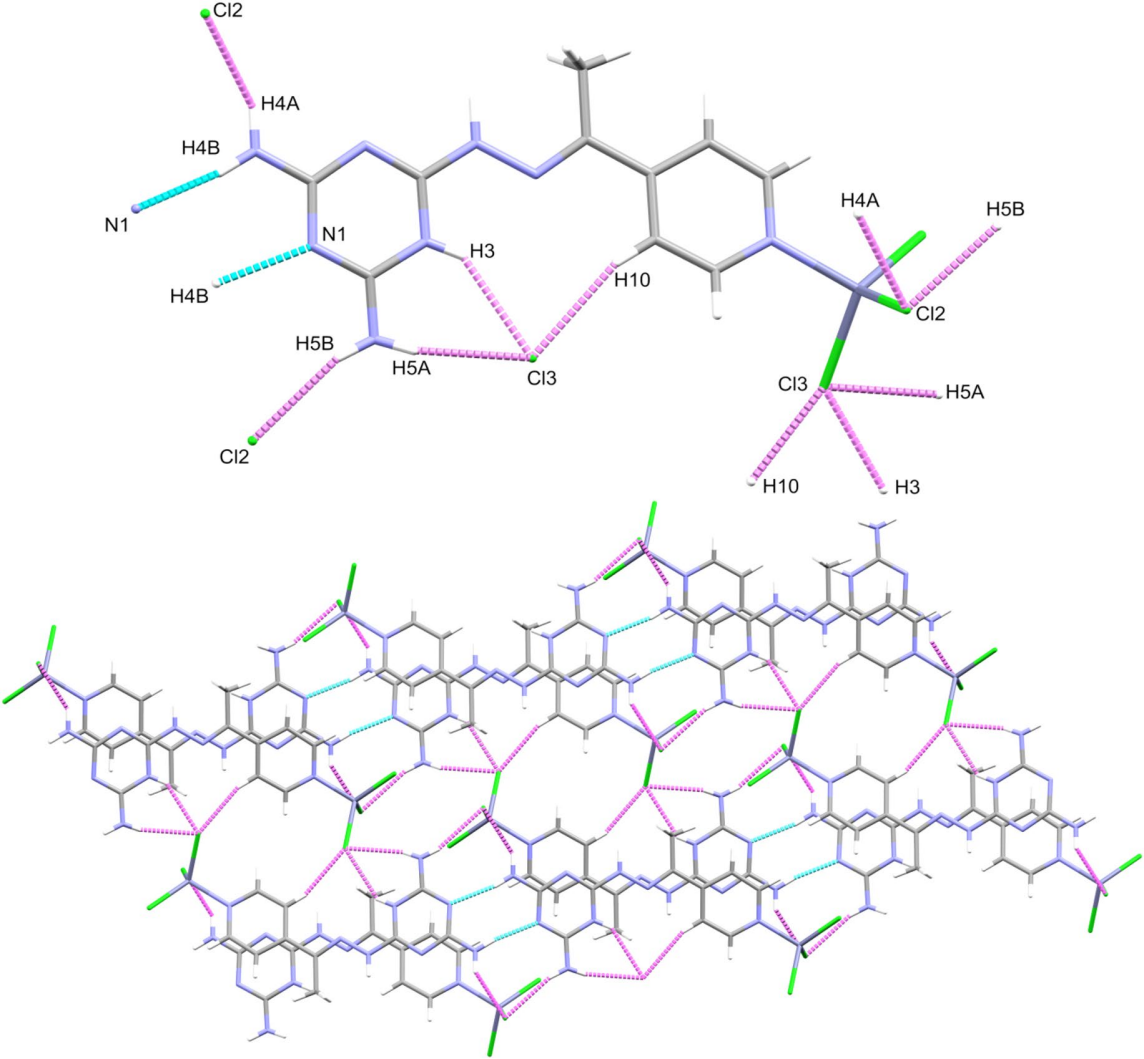
Hydrogen bond contacts (**upper**) and crystal packing (**lower**) of **1**. The purple and turquoise dotted lines refer to Cl···H and N···H interactions, respectively.

The Zn-N_(pyridine)_ bond distance is 2.050(2) Å, while the Zn-Cl bond distances range from 2.2264(7) to 2.2759(6) Å. The Zn–N and Zn–Cl bond lengths are very close to those of the complexes previously reported by Yu et al.^16^, and Smolková et al.^43^. On the other hand, the four donor atoms form bond angles range from 102.91(6) to 115.43(3)º (Table 1), where the smallest and largest angles are N8-Zn1-Cl3 and Cl1-Zn1-Cl2, respectively, deviating from the ideal value of 109.5º, indicating a distortion from the conventional tetrahedral geometry. Additionally, the τ_4_^44^ and τ_4_′^45^ geometric indices, which assess the degree of distortion in tetra-coordinated structures, are estimated to be 0.93 and 0.92, respectively, further confirming this slight distortion Fig. 1B. The τ_4_ value is comparable to that of the [Zn(HL)Cl_3_] complex (L = 1-(4-(1*H*-Imidazol-1-yl)benzyl)-1*H*-1,2,4-triazole), which has a τ_4_ value of 0.94 ^16^. On the other hand, the crystal packing of **1** is stabilized by one N-H···N and a number of N-H···Cl intermolecular hydrogen bonds besides C-H···Cl interactions as illustrated in Fig. 2. All the hydrogen bond specifications are found in Table S2. For the N-H···N hydrogen bond, the interaction takes place between the N_(*s-*triazine)_ and the hydrogen atom of one of the two amino groups, with an H···N distance of 2.10 Å for the N4-H4B···N1. The respective donor (D)···acceptor (A) distance is 2.953(3) Å. The C-H···Cl interactions occurs between the pyridyl C-atoms (C10) acting as the hydrogen bond donor and the coordinated Cl3 atom as the hydrogen acceptor, with a donor-acceptor distance of 3.534(3) Å. In contrast, the N-H···Cl interactions are in the range of 2.45 to 2.58 Å for N4-H4A···Cl2 and N5-H5B···Cl2, respectively.

**Table 1:** Chosen bond distances (Å) and angles (º) of **1**.

Bond	Length	Bond	Length
Zn1-Cl1	2.2264(7)	Zn1-Cl3	2.2759(6)
Zn1-Cl2	2.2661(7)	Zn1-N8	2.050(2)

Bonds	Angle	Bonds	Angle
Cl1-Zn1-Cl2	115.43(3)	N8-Zn1-Cl1	107.39(6)
Cl1-Zn1-Cl3	113.90(3)	N8-Zn1-Cl2	111.04(6)
Cl2-Zn1-Cl3	105.51(2)	N8-Zn1-Cl3	102.91(6)

**Fig. 3:**
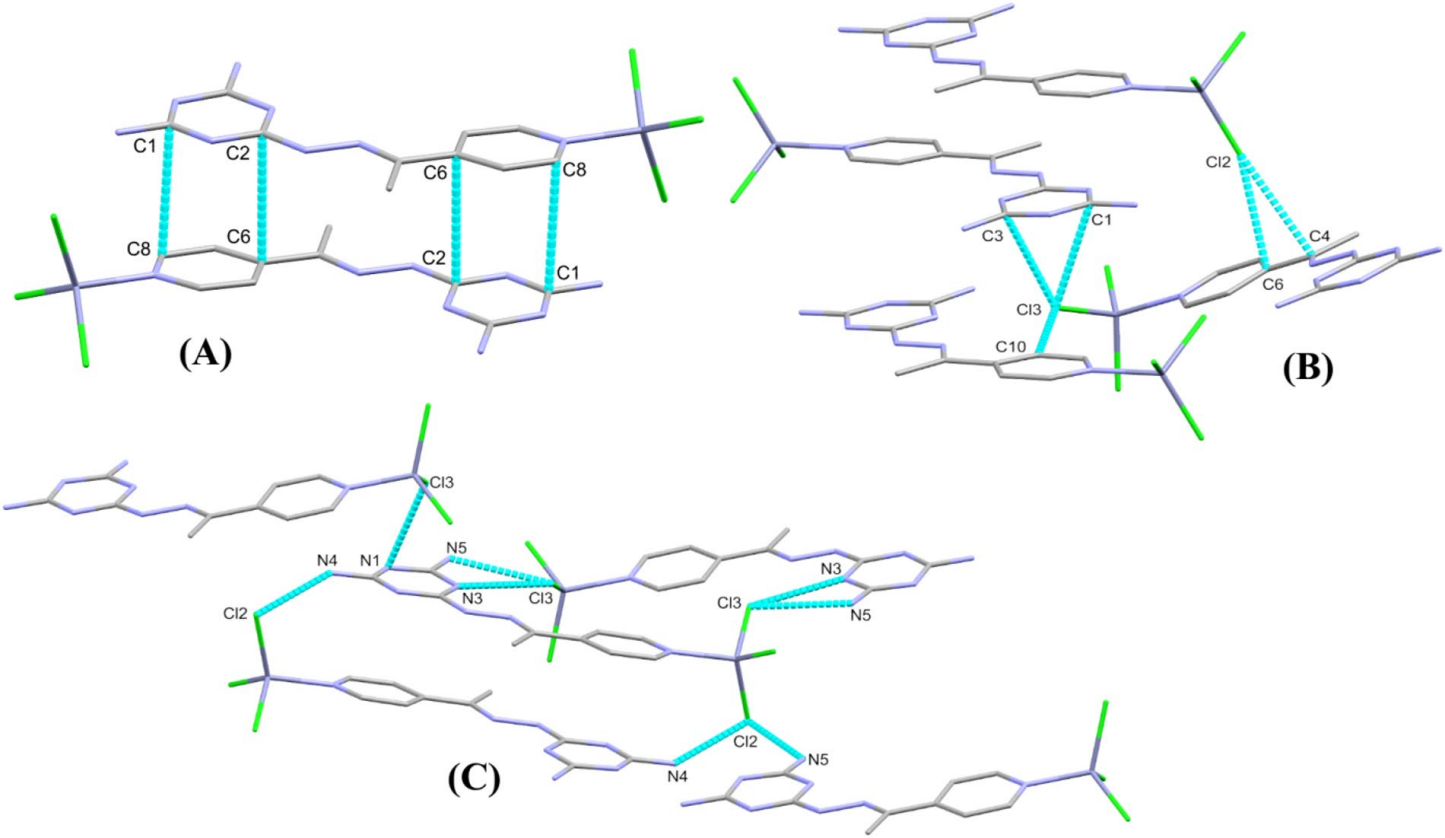
π···π stacking (**A**), anion···π contacts (**B** and **C**) of **1**. Hydrogen atoms have been excluded for better visibility.

Moreover, π···π stacking connections between *s-*triazine and pyridine rings with distances equal to 3.322 and 3.324 Å for C1···C8 and C2···C6, respectively, further strengthen the crystal structure of complex **1** (Fig. 3A). The centroid-to-centroid separation is 3.635 Å.

It’s also important to point out that there are a significant number of anion···π contacts were observed in **1** as shown in Fig. 3. All details regarding these short contacts are depicted in Table S3. For the Cl···C interactions shown in Fig. 3B, the shortest distances are between the chloride anion and C_(*s-*triazine)_, specifically Cl3···C3 (3.316 Å) and Cl3···C1 (3.409 Å). In addition, there are five Cl···N interactions (Fig. 3C), the shortest interactions are Cl2···N4_(amino)_, Cl3···N3_(*s-*triazine)_, and Cl3···N5_(amino)_, with distances of 3.180, 3.240, and 3.254 Å, respectively.

#### Structure of [Ag_4_(HL)_2_(NO_3_)_6_]_n_ complex (**2**)

**Fig. 4:**
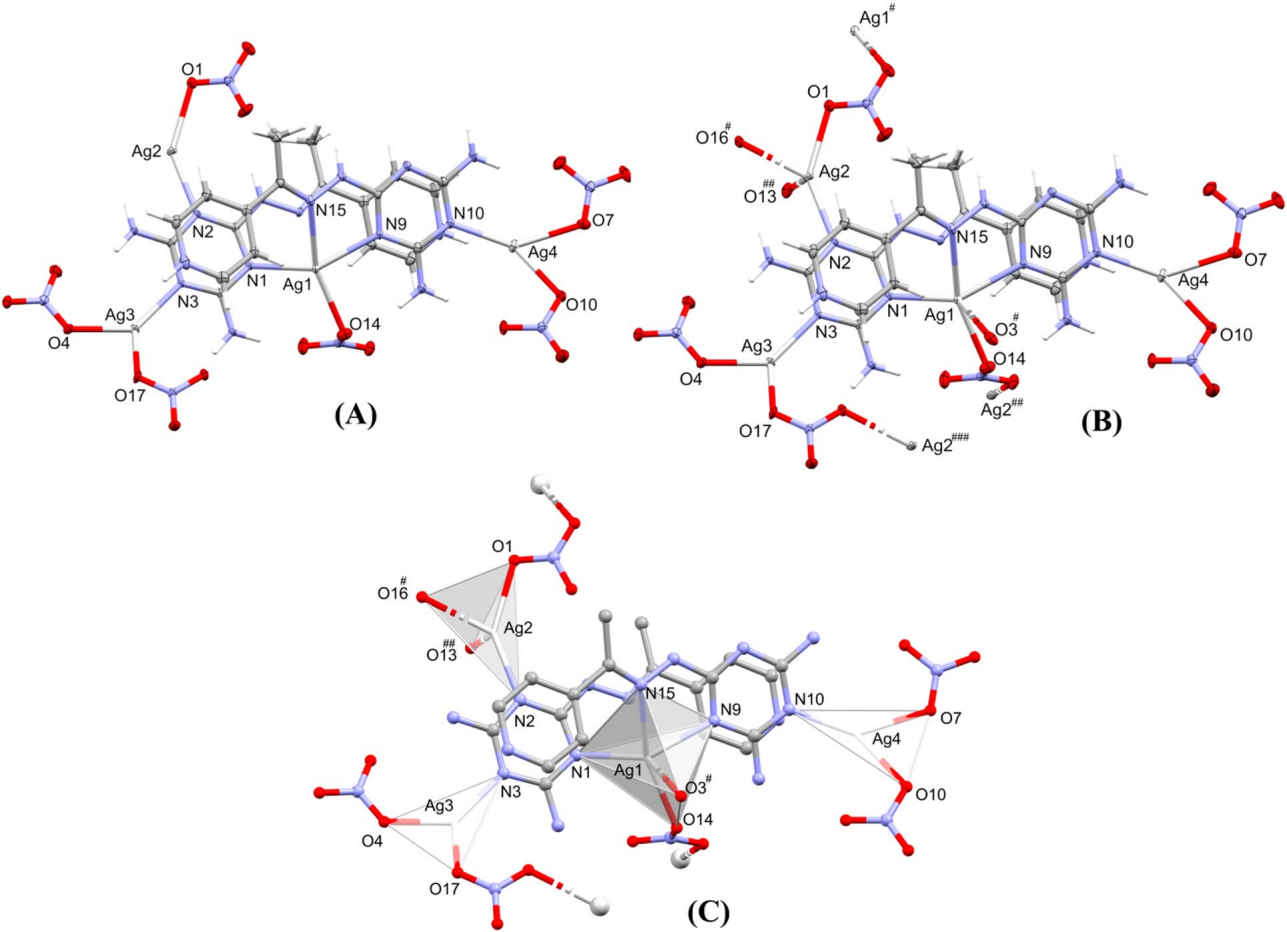
X-ray structure illustrating asymmetric unit (**A**), the complete coordination environments around Ag(I) centers (**B**) and the distorted geometries of [Ag_4_(HL)_2_(NO_3_)_6_]_n_ complex **2** (**C**). ORTEP diagram, illustrating thermal ellipsoids at 50% probability level, is shown in Fig. S11. Symm. codes ^#^ -1+x,y,z; ^##^ -x,2-y,1-z; ^###^ 1+x,y,z.

Complex **2** (CCDC 2518342) crystallizes in the triclinic crystal system with space group *P-1* (Table S1) showing interesting 3D polymeric. Unlike complex **1**, both the organic ligand units in **2** are protonated at the nitrogen atom of the pyridyl moiety leading to the cationic **HL**^**+**^ ligand formula. Hence, all the *s*-triazine N-atoms are involved in the coordination with the four crystallographically non-equivalent Ag(I) ions which allowed the polymer expansion in case of **2**. As a result, the asymmetric unit of **2** is completed by six nitrate groups to balance the net charge, leading to the neutral tetranuclear [Ag_4_(HL)_2_(NO_3_)_6_] (Fig. 4A). As illustrated in this figure, the two organic ligands are parallel but oriented in opposite directions. Specifically, the *s-*triazine ring in one organic ligand unit is parallel to the pyridine ring in the other **HL**^**+**^ unit. Figs. 4B and 4C showed the complete coordination environments and different geometries, respectively, around the four crystallographically independent Ag(I) ions in **2**.

In Ag(I) complexes, various geometries often coexist within a single structure, such as mixed coordination numbers of 2 and 4^46^, 3 and 4^47^, or 6 and 7^48^. However, our Ag(I) complex features a combination of 3, 4, and 5 coordination numbers (Fig. 4B). Some bond distances and angles for **2** are provided in Table 2. The first silver ion (Ag1) is coordinated by two N-atoms from the *s-*triazine (N9) and azomethine (N15) moieties of one organic ligand with bond lengths of 2.340(3) and 2.565(3) Å, respectively, while the bite angle N9-Ag1-N15 is 67.56(9)º. In addition, there is a slightly short Ag1-N1 bond with the *s-*triazine moiety from another **HL**^**+**^ ligand (Ag1-N1; 2.324(3) Å). The penta-coordination environment around Ag1 is completed by two O-atoms (O3^#^ and O14), ^#^-1+x,y,z, from two nitrate ions. Thus, based on the trigonality index proposed by Addison^49^, the geometry around Ag1 metal center is described as distorted square pyramidal (τ_5_ = 0.23).

The Ag2 center is tetra-coordinated being bonded to one *s-*triazine N-atom from **HL**^**+**^ ligand, in addition to three oxygen atoms from three different nitrate groups. The angles around the metal center range from 73.98(8) to 142.94(9)º (Table 2). Additionally, the values of the τ_4_ and τ_4_ˊ geometric indices, are estimated to be 0.55 and 0.54, respectively. Hence, the coordination geometry around Ag2 could be described as an intermediate between square planar and tetrahedron (Fig. 4C). Regarding Ag3 and Ag4, both are tri-coordinated with one nitrogen atom from *s*-triazine in addition to two oxygen atoms from two nitrate groups. The Ag3 and Ag4 silver centers exhibit a distorted trigonal planar geometry with angles range from 75.46(8)º to 137.07(9)º and from 68.15(8)º to 143.19(9)º for Ag3 and Ag4, respectively (Table 2). It has been noted that, all Ag–N_(*s*-triazine)_ bond lengths in **2** are shorter than that reported for [Ag(BPAT)(ACN)]ClO_4_^50^ complex (2.368(3) Å), where, BPAT = 2-amino-4,6-*bis*(3,5-dimethyl-1*H*-pyrazol-1-yl)-1,3,5-triazine.

**Table 2:** Coordinate bond lengths (Å) and angles (º) of **2**

Bond	Length	Bond	Length
N1-Ag1	2.324(3)	O14-Ag1	2.437(2)
N2-Ag2	2.264(3)	O17-Ag3	2.410(2)
N3-Ag3	2.206(3)	O13-Ag2^i^	2.534(3)
O4-Ag3	2.308(2)	N15-Ag1	2.565(3)
O1-Ag2	2.359(2)	O16-Ag2^ii^	2.455(2)
O7-Ag4	2.399(3)	Ag2-O13^i^	2.534(3)
N9-Ag1	2.340(3)	Ag2-O16^iii^	2.455(2)
O10-Ag4	2.352(2)	Ag4-Ag4^iv^	3.2426(5)
N10-Ag4	2.201(3)		

Bond	Angle	Bond	Angle
N1-Ag1-N9	144.65(9)	O16^iii^-Ag2-O13^i^	73.98(8)
N1-Ag1-O14	105.62(9)	N3-Ag3-O4	137.07(9)
N1-Ag1-N15	95.66(9)	N3-Ag3-O17	127.92(9)
N9-Ag1-O14	95.98(9)	O4-Ag3-O17	75.46(8)
N9-Ag1-N15	67.56(9)	O7-Ag4-Ag4^iv^	57.88(6)
O14-Ag1-N15	158.36(9)	O10-Ag4-O7	68.15(8)
N2-Ag2-O1	142.94(9)	O10-Ag4-Ag4^iv^	61.84(6)
N2-Ag2-O13i	99.93(9)	N10-Ag4-O7	143.19(9)
N2-Ag2-O16^iii^	138.90(9)	N10-Ag4-O10	141.92(9)
O1-Ag2-O13^i^	92.72(9)	N10-Ag4-Ag4^iv^	143.85(7)
O1-Ag2-O16^iii^	78.07(8)		

Symm. code ^i^-x,2-y,1-z; ^ii^1+x,y,z; ^iii^-1+x,y,z; ^iv^1-x,1-y,2-z.

**Fig. 5:**
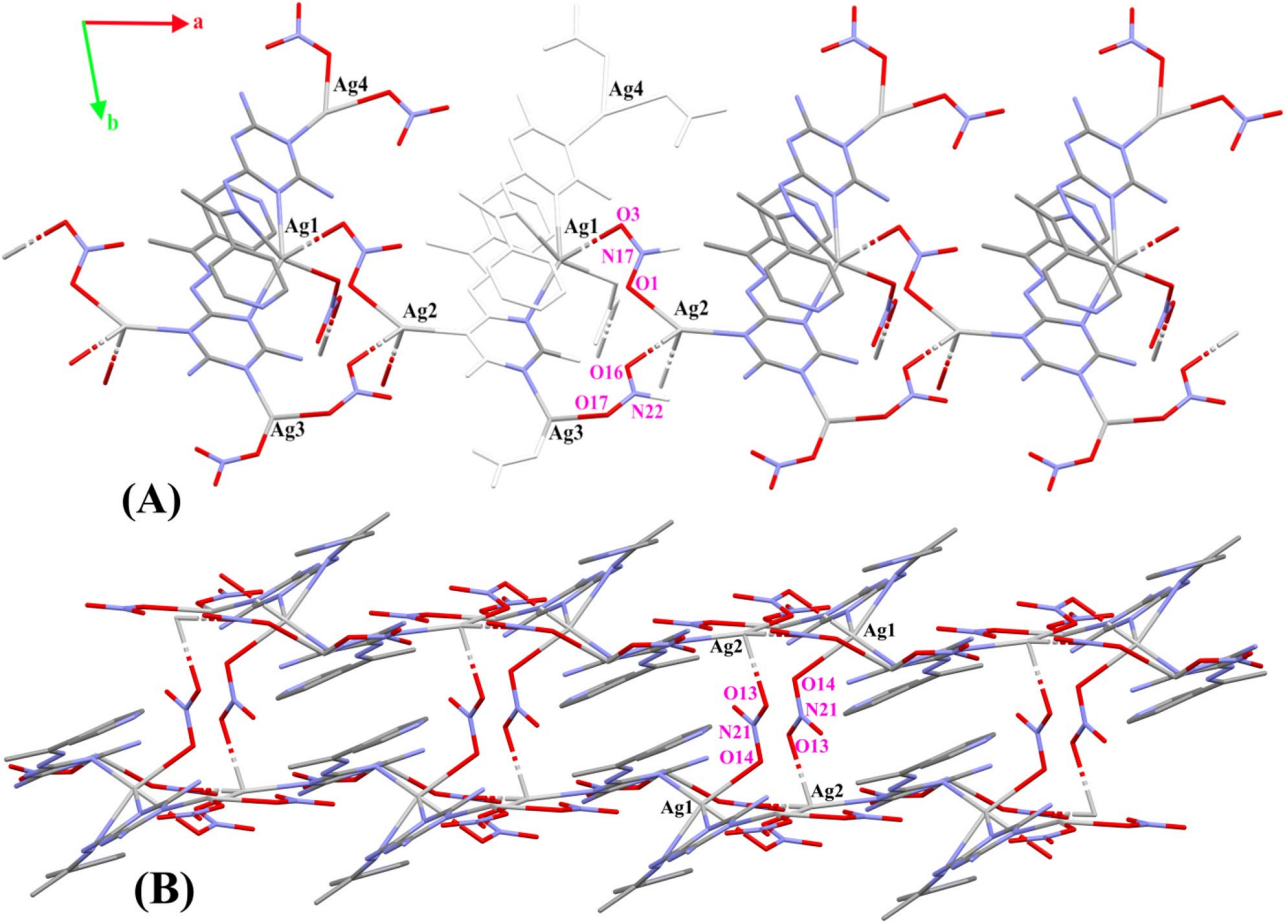
The extension of the infinite coordination polymer [Ag_4_(HL)_2_(NO_3_)_6_]_n_ along *a*-axis (**A**) and the extended array along the same axis (**B**). All H-atoms are omitted for clarity.

The 3D polymeric structure configuration of **2** is constructed *via* the coordination interactions between Ag(I) with both ligand groups (NO_3_^−^ and **HL**^**+**^). Specifically, the *s-*triazine ring in one **HL**^**+**^ ligand forms the three coordinate bonds Ag1-N1, Ag2-N2, and Ag3-N3 which participate in the formation of the polymeric chains. Additionally, Ag2 from one unit is linked to Ag1 and Ag3 from another adjacent unit *via* two nitrate groups. Ag2 is bonded to Ag1 of another adjacent unit through Ag2-O1-N17-O3-Ag1 bridge, while Ag2 is bonded to Ag3 *via* Ag2-O16-N22-O17-Ag3 bridge as shown in Fig. 5A leading to a twelve-membered ring. As a result, infinite polymeric chain along the *a*-axis is formed, as illustrated in Fig. 5A. Furthermore, each two of these 1D infinite polymeric chains are oriented anti-parallel to each other, which enhances further connection *via* the Ag1-O14-N21-O13-Ag2 bridge. These results in the formation of an array extended through the *a*-direction (Fig. 5B).

**Fig. 6:**
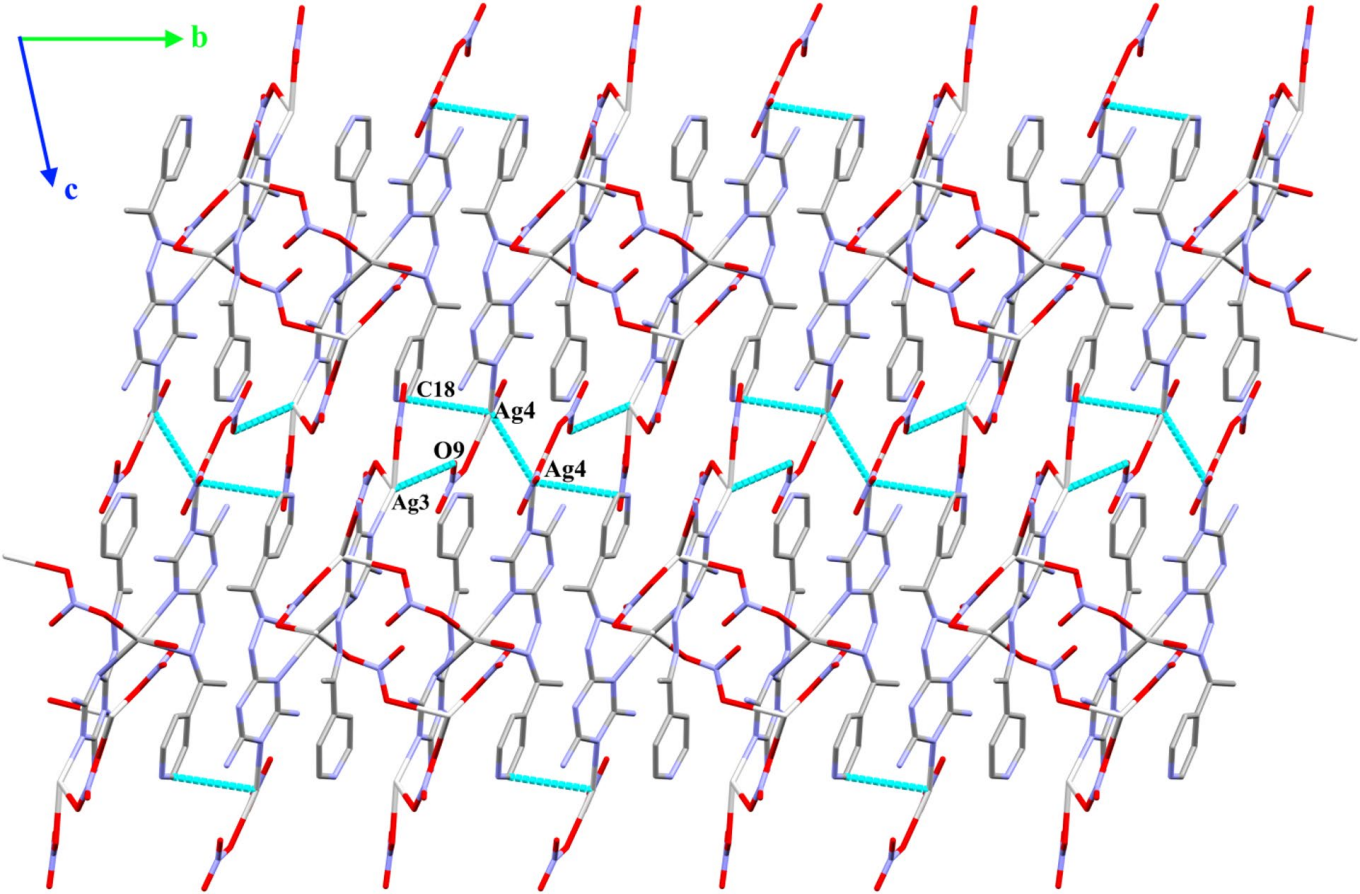
Infinite polymeric chains of **2** along *b* and *c*-directions. All H-atoms are removed to enhance clarity.

Then through relatively strong Ag3···O9 (2.772 Å) and argentophilic Ag4···Ag4 (3.242 Å) contacts, the infinite polymeric chains along *b* and *c* directions are formed as displayed in Fig. 6. Moreover, Ag4···C18 (3.391 Å) contact enhances the polymeric extension along *b*-axis (Fig. 6). As a result, 3D coordination polymer of **2** is formed.

Moreover, the crystal packing of **2** was stabilized by a large number of intermolecular hydrogen bonds forming a 3D net structure (Fig. S12). This large number of hydrogen bonds is mainly a result of the significant number of NO_3_^−^ and NH_2_ groups within the coordination sphere of **2**. In these interactions, the O-atoms of the NO_3_^−^ groups act as hydrogen bond acceptor, while the hydrogen bond donors can be either nitrogen or carbon, as shown in Figs. S12A and S12B, respectively, and detailed in Table S4. When the protonated pyridine N-atom acts as the H-bond donor, the D-A distances are shorter (2.774(4) and 2.714(4) Å for N8-H8···O10 and N16-H16···O17, respectively) than those observed when the amino group serves as H-bond donor (D···A lengths ranged from 2.875(4) to 3.072(4) Å) as depicted in Table S4. On the other hand, the C-H groups from the methyl or pyridyl rings are the H-bond donor for the C-H···O contacts, as detailed in Fig. S12B and Table S4.

**Fig. 7:**
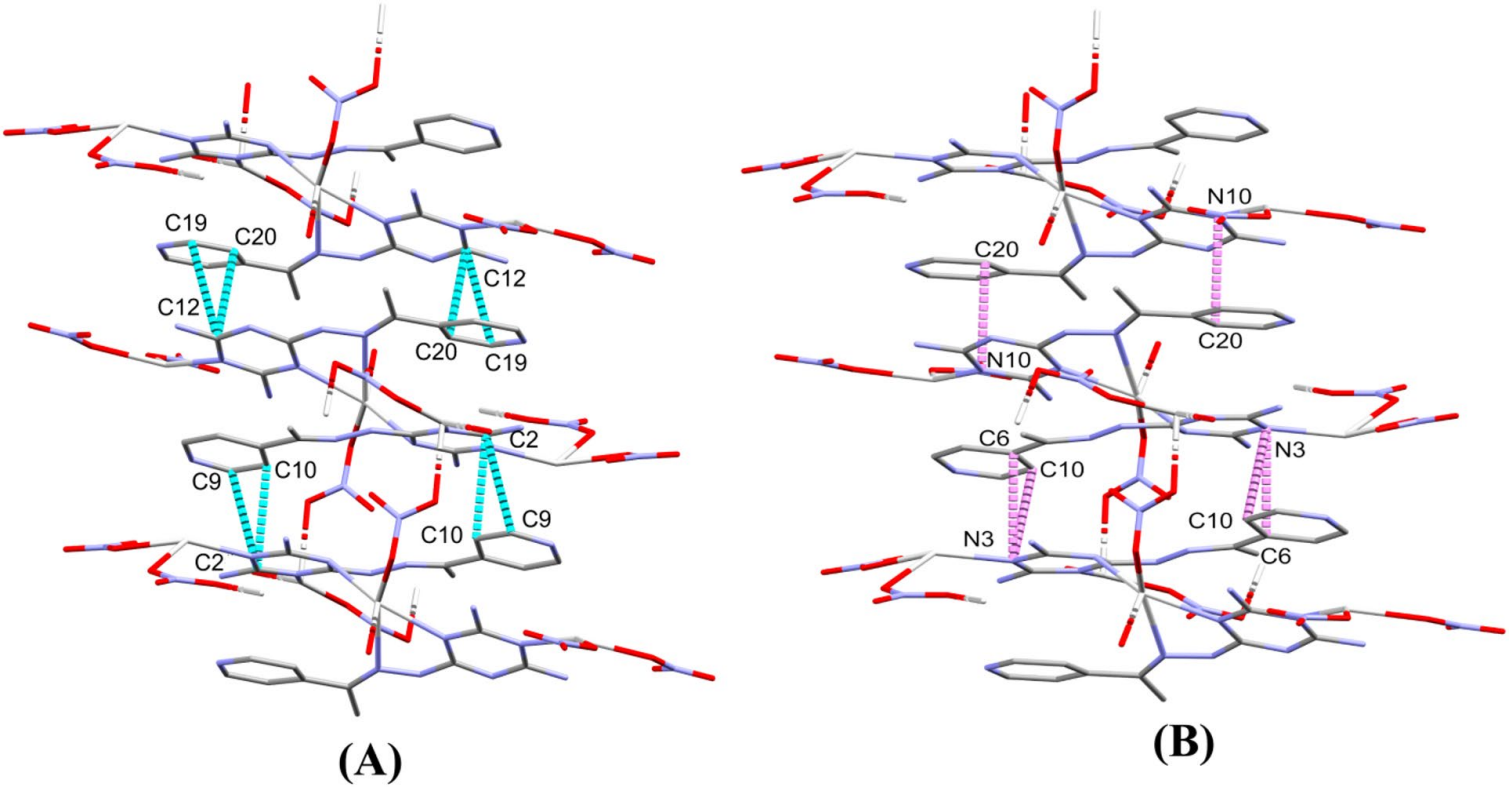
π···π stacking interactions showing the C···C (**A**) and C···N (**B**) contacts of **2**. All H-atoms are removed to enhance clarity.

It is observed that, effective π···π stacking contacts between the *s-*triazine and pyridine moieties of **HL**^**+**^ are important in the molecular packing of **2** (Fig. 7). These interactions involve carbon-to-carbon and carbon-to-nitrogen contacts. As illustrated in Fig. 7, these stacking interactions occur both within and between the arrays extending along the *a*-direction. All details of these contacts are depicted in Table S5. The C12···C20 (3.211Å), C12···C19 (3.300Å), and N10···C20 (3.403Å) interactions occur between the arrays, while the other interactions take place within them (Fig. 7).

**Fig. 8:**
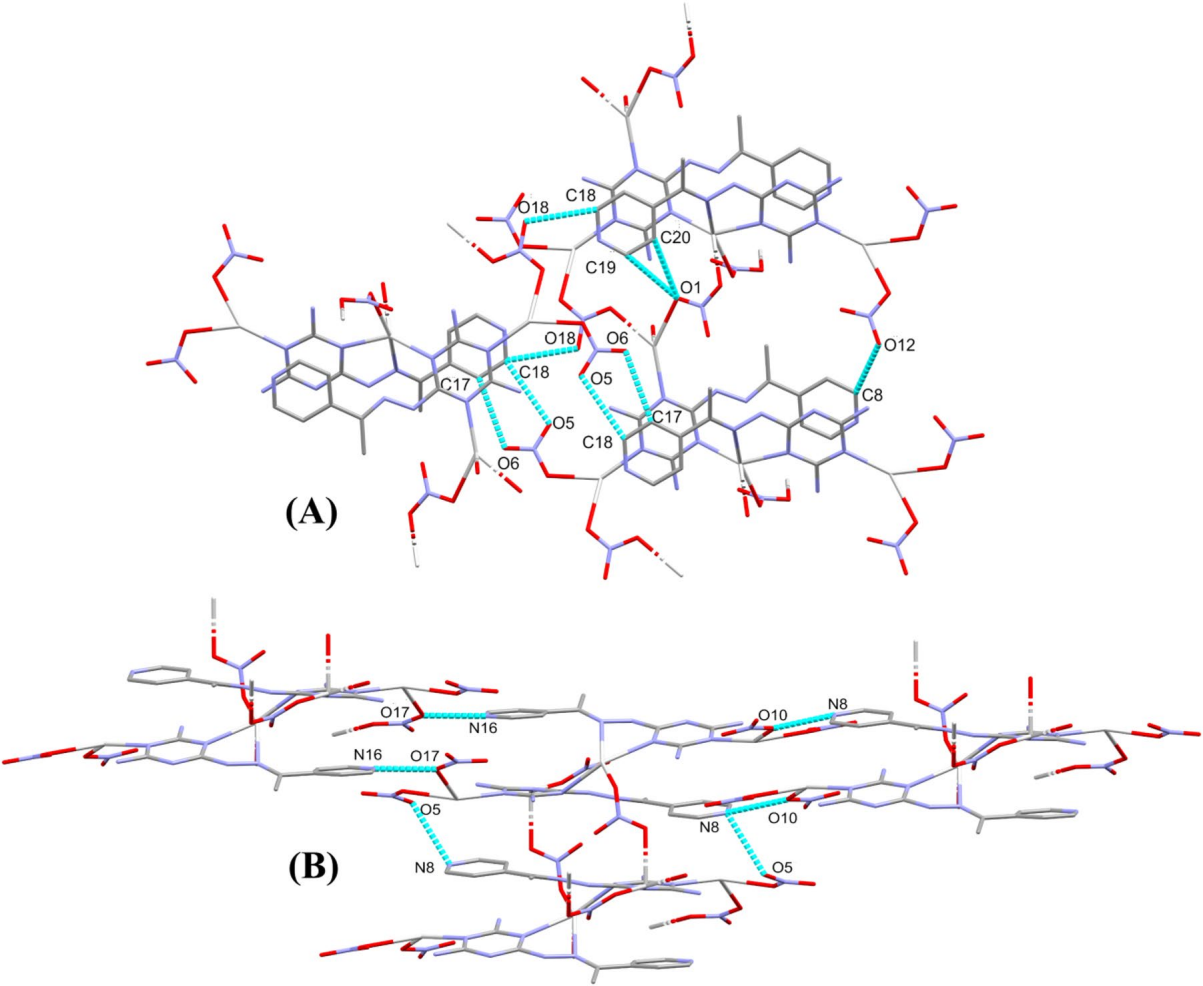
Anion···π stacking: O···C (**A**) and O···N (**B**) in **2**. All H-atoms are excluded for better visibility.

It is noteworthy that there are a significant number of anion···π stacking interactions between the oxygen atoms of the NO_3_^−^ groups with the C- and N- atoms of the π-system from the *s*-triazine and pyridine moieties (Fig. 8). The O1···C20 (3.160 Å) and O17···N16 (2.714) contacts (Table S5). Further insights into the packing and intermolecular interactions in both complexes can be gained through Hirshfeld analysis.

### Hirshfeld surfaces

**Fig. 9:**
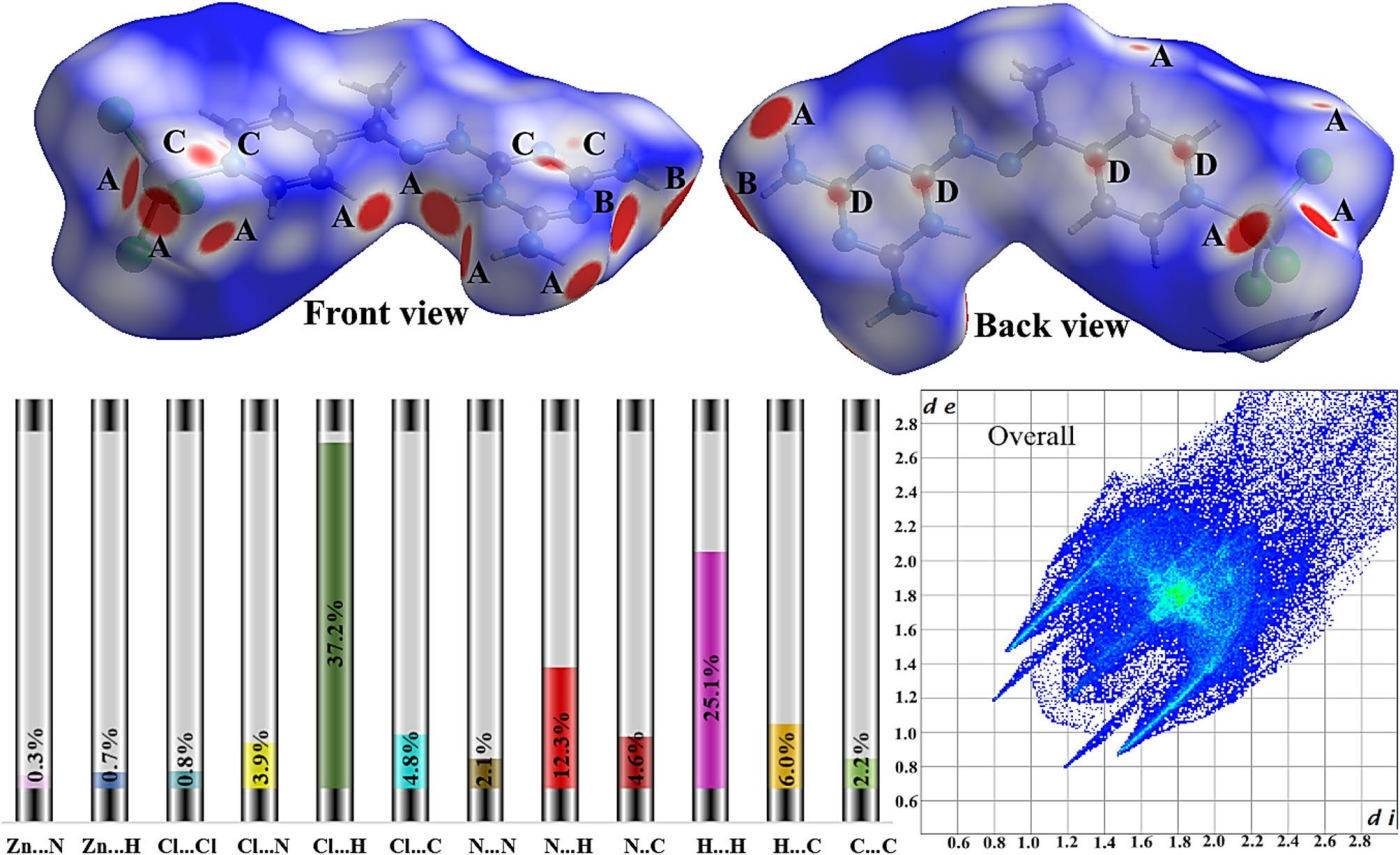
Full d_norm_ map (upper) shows significant contacts: (**A**) Cl···H, (**B**) N···H, (**C**) Cl···C, and (**D**) C···C, while the contributions of various interactions and the overall fingerprint plot are illustrated (lower) for complex **1**.

Hirshfeld analysis is studied to enhance our understanding of the supramolecular structure. It provides both qualitative and quantitative insights into the types of intermolecular contacts involved in the crystal packing. The analysis was performed on the asymmetric unit of both complexes. In [Zn(HL)Cl_3_] complex (**1**), the full 3D d_norm_ map and overall 2D fingerprint plot of the all interactions are shown in Fig. 9. The analysis reveals several prominent dark red areas in the d_norm_ map of complex **1** (Fig. 9), indicating strong interactions, particularly between the coordinated Cl^−^ anion and hydrogen atoms. This is supported by sharp spikes in the fingerprint plots (Fig. S13), highlighting the Cl···H interaction as the most significant for the crystal packing of complex **1**. Additionally, the N···H interaction is also notable, appearing as intense red spots (Fig. 9) and sharp spikes (Fig. S13), emphasizing its role in the supramolecular structure. Other critical interactions include Cl···C and C···C, which enhance the crystal stability of **1**. In terms of molecular packing contributions, Cl···H interactions account for 37.2%, followed by H···H at 25.1% and N···H at 12.3% (Fig. 9). Additional contacts are observed in the crystal structure of **1**; however, they are less significant in the molecular packing due to their longer interaction distances and are represented in Fig. 9.

**Fig. 10:**
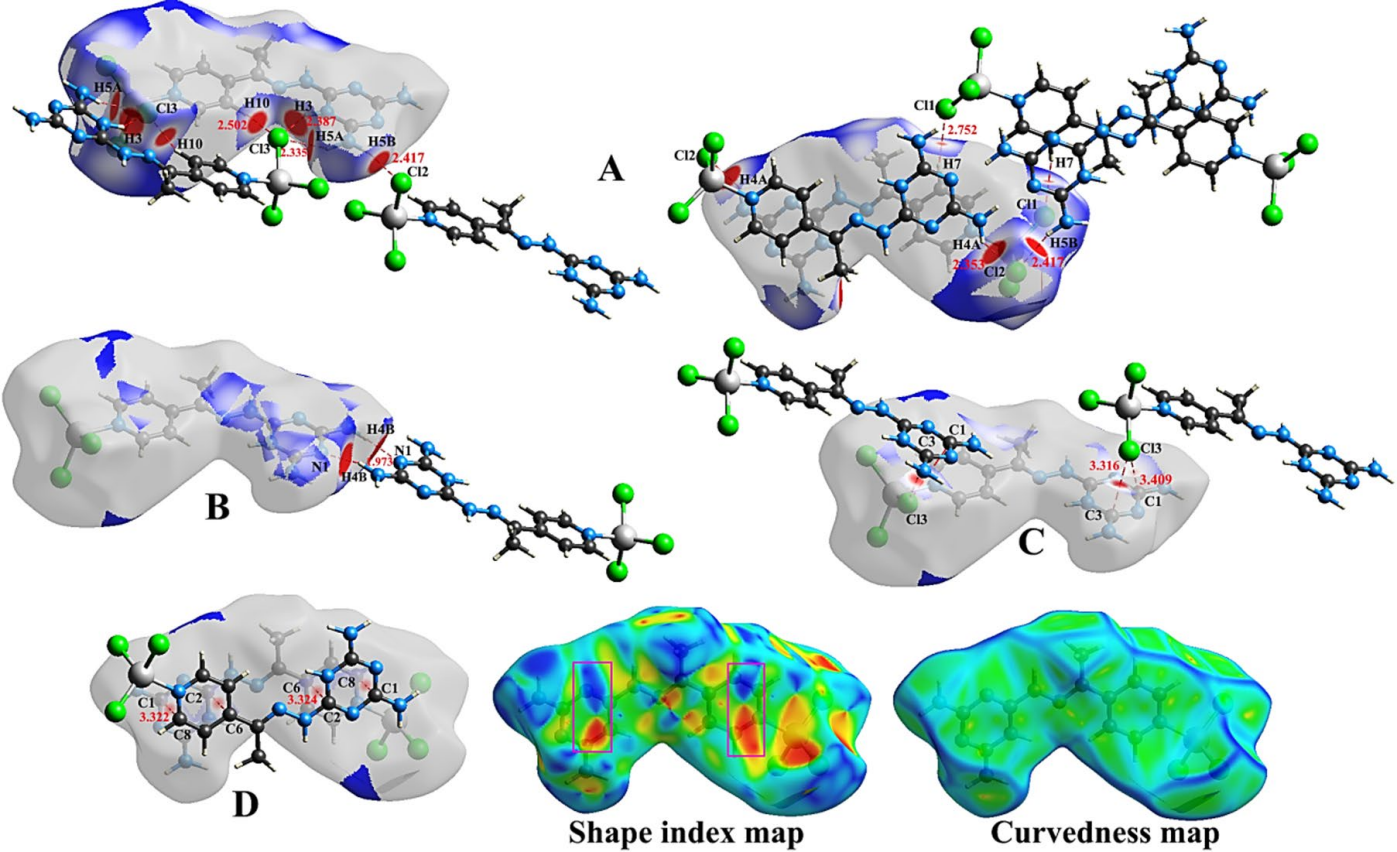
Presentation of the shortest intermolecular interactions for Cl···H (**A**), N···H (**B**), Cl···C (**C**), and π···π stacking (**D**). The π···π stacking interactions also appeared as red/blue triangles in the shape index, and a flat green area in the curvedness map (lower). All contact distances are in angstroms.

The Cl···H, N···H, and Cl···C short contacts are illustrated in Fig. 10, along with their respective distances. The Cl···C interactions are due to the anion···π stacking interactions between the coordinated chloride ion and the carbon atoms of the *s*-triazine moieties in the organic ligand (**HL**^**+**^) as shown in Fig. 10C.

Moreover, C···C interaction arises from π···π stacking between the C_(*s*−triazine)_ and C_(pyridine)_ are observed as red zones in the d_norm_ map (Fig. 10D). The red/blue triangles seen in the shape index map, along with the green flat areas on the curvedness map, further confirm the existence of aromatic π···π stacking contacts in **1** (Fig. 10). This is in agreement with what was mentioned in the crystal structure analysis of [Zn(HL)Cl_3_] complex (**1**).

**Fig. 11:**
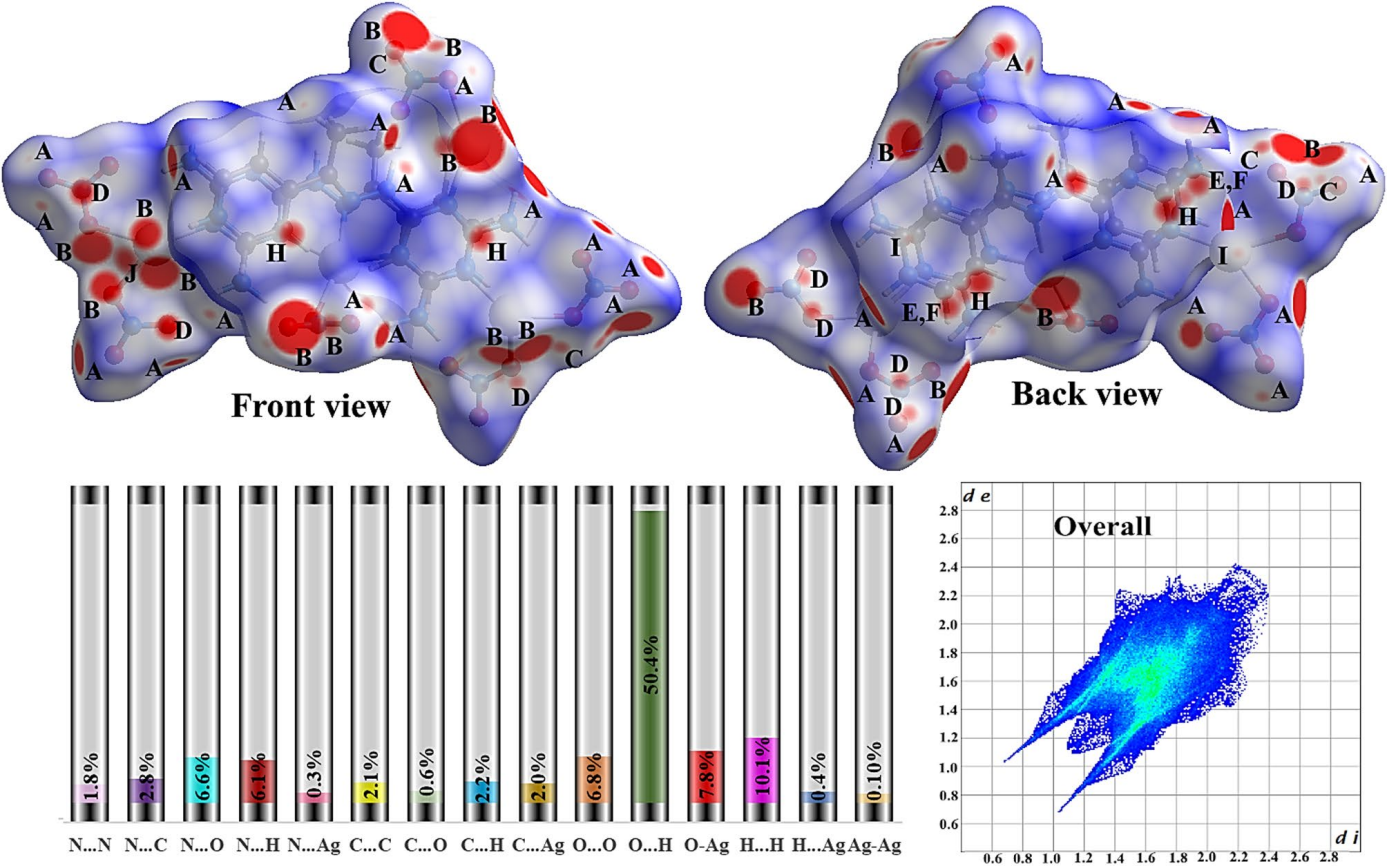
Full d_norm_ map (upper) shows the most significant contacts: (**A**) O···H, (**B**) O-Ag, (**C**) O···O, (**D**) N···O, (**E**) N···H, (**F**) N···C, (**H**) C···C, (**I**) C···Ag, and (**J**) Ag···Ag., while the contributions of various interactions and the overall fingerprint plot are illustrated (lower) for complex **2**.

For [Ag_4_(HL)_2_(NO_3_)_6_]_n_ complex (**2**), the full d_norm_ map and the overall 2D fingerprint plot are illustrated in Fig. 11. The polymeric Ag-O interactions (7.8%) displayed as deep red areas (**B**) in the d_norm_ map (Fig. 11) and also as two distinct sharp spikes in the fingerprint map (Fig. S14), indicating that the coordination interactions between the Ag(I) and the O-atoms of the coordinated NO_3_^−^ anions shows an important role in the extension of the 3D polymeric backbone of **2**.

In addition, the argentophilic (Ag4···Ag4 = 3.242 Å) contact, which enhances the infinite polymeric chains along *b* and *c* directions, as discussed in X-ray description, appears as a red spot (J) in the d_norm_ map (Fig. 11). Moreover, O···H hydrogen bonding interactions significantly contribute to the crystal stability of **2**, as it appears as red spots (A) in the d_norm_ map (Fig. 11). This is also supported by the presence of two sharp spikes in its corresponding fingerprint plot (Fig. S14). This interaction represents the majority of the total Hirshfeld surface area (50.4%) followed by H···H interaction (10.1%) as shown in Fig. 11.

Interestingly, the C···C (2.1%) and O···N (6.6%) contacts reveal the value of the π···π and anion···π stacking contacts, respectively. In the former, this interaction occurs between the C_(*s-*triazine)_ and C_(pyridine)_ of the organic ligand (**HL**^**+**^) and confirmed by green flat surface patches in the curvedness map (Fig. S15). This figure illustrates the representation of this interaction as red dotted lines, in addition to their lengths.

The hydrogen-based interactions played a predominant role in their molecular packing. Comparing the two complexes, the interactions involving hydrogen atoms are more prevalent in complex **1** (81.3%) than **2** (69.2%). This is attributed to the 3D polymeric structure of **2**, characterized by the presence of a proportion of coordinated contacts of Ag-O, Ag···C, as well as the argentophilic Ag···Ag interaction, which are responsible for the infinite polymeric extension of **2**. Additionally, the propensity of specific atom pairs to form contacts within the crystal could be predicted using the enrichment ratio (E_XY_) calculations.

#### Enrichment ratio (E_XY_) analysis

The surface contact percentages obtained from Hirshfeld analysis of complexes **1** and **2** are utilized to calculate enrichment ratio (E_XY_)^51^, which help assess the tendency of atom pairs to form contacts within a crystal. E_XY_ values greater than one indicates that these pairs have a strong tendency to form interactions within the crystal structure, whereas pairs with E_XY_ values less than unity display a low prospect to interact. The calculated E_XY_ values for the two studied complexes are tabulated in Table S6. In complex **1**, The E_XY_ values greater than 1 are observed for the Zn···N, Zn···H, Cl···H, Cl···C, N···N, N···C, and C···C interactions. In **2**, the E_XY_ ˃1 for the N···N, N···C, C···C, C···Ag, O···H, and O···Ag contacts. These findings highlight the increased probability of interactions occurring between these atom pairs. Conversely, the rest of interactions showed lower ability to form intermolecular interactions as shown in Table S6. Notably, the high E_XY_ value of 6 for the C···C contact in the polymeric silver complex **2** indicates an exceptionally strong preference for this interaction.

### NBO study

#### Natural charge determination

The charges at the metal center and its surrounding ligands were analyzed using natural charge calculations^52^ at the X-ray geometry. The ωB97XD method was employed to quantify the charge transfer from the organic ligand and anions as Lewis bases to the metal ion as a Lewis acid resulting from complexation. The resulting net natural charges at the metal ions and different ligands for both complexes are depicted in Table 3. In the [Zn(HL)Cl_3_] (**1**), there is a significant decrease in the charge of the Zn(II) to 0.9916 e. In this complex, the Zn(II) is coordinated by one protonated ligand (**HL**^**+**^) and three chloride anions. As a result, there are 1.0084 e transferred from these ligands to Zn(II). Furthermore, **HL**^**+**^ and the three chloride anions reduce the +ve charge on the Zn(II) ion by 0.0874 and 0.921 e, respectively. In the 3D polymeric [Ag_4_(HL)_2_(NO_3_)_6_]_n_ complex (**2**), each crystallographically non-equivalent Ag(I) ion is analyzed individually, considering its full coordination environment. The calculated natural charges of the Ag(I) center in these units are 0.5657, 0.5518, 0.6223, and 0.6047 e for Ag1, Ag2, Ag3, and Ag4 unit, respectively, instead of + 1.0000 for the free silver ion. The natural charge on the silver atom in Ag1 and Ag2 units is lower than that in Ag3 and Ag4 units. This is attributed to a higher coordination number and the larger number of coordination anions in the former compared to the latter.

**Table 3:** The net natural charges at the metal ions, the coordinated anions and **HL**^**+**^ ligands for complexes **1** and **2** using ωB97XD methods.

Complex 1		Complex 2				
			Ag1 unit	Ag2 unit	Ag3 unit	Ag4 unit
Atom	Charge	Atom	Charge			
Zn	0.9916	Ag	0.5657	0.5518	0.6223	0.6047
**HL**	1.0874	**HL**	2.1417^a^	1.0214	1.0680	1.0688
3Cl	− 2.0790	NO_3_	− 1.7074^a^	− 2.5732^b^	− 1.6903^a^	− 1.6735^a^

^a^ two, ^b^ three of the corresponding ligand.

Table 3 summarizes the effects of **HL**^**+**^ and nitrate groups on the positive charge reduction across different Ag units. In the first Ag unit, the two **HL**^**+**^ and the two nitrate groups reduce the Ag(I) charge by 0.1417 and 0.2926 e, respectively. In the Ag2 unit, these values are 0.0214 and 0.4268 e from one **HL**^**+**^ and three nitrate groups respectively. In the Ag3 and Ag4 units, the organic ligand transported 0.0680 e and 0.0688 e to the Ag(I) ion, respectively, while the two coordinated nitrate groups transferred 0.3097 e and 0.3265 e, respectively. In addition, the orbital–orbital interactions for the coordinated bonds around central metal ions were conducted using NBO interactions.

#### NBO interactions

The strength of contacts between the metal ion and various ligand molecules is evaluated by calculating the interaction energy (E^(2)^) based on second-order perturbation theory. All interactions between different orbitals involved in the coordinate bonds around Zn(II) and Ag(I) in complexes **1** and **2**, respectively, are depicted in detail in Table S7. In complex **1**, the Zn-Cl coordination interactions have very high interaction energy which indicates strong interaction with the coordinated chloride anions. The E^(2)^ values are 100.02 kcal/mol (LP(4)Cl2→LP*(7)Zn1), 88.43 kcal/mol (LP(4)Cl3→LP*(6)Zn1), and 77.64 kcal/mol (LP(4)Cl4→ LP*(6)Zn1). Also, the Zn1-N8 interaction has a relatively high E^(2)^ value (49.75 kcal/mol).

**Fig. 12:**
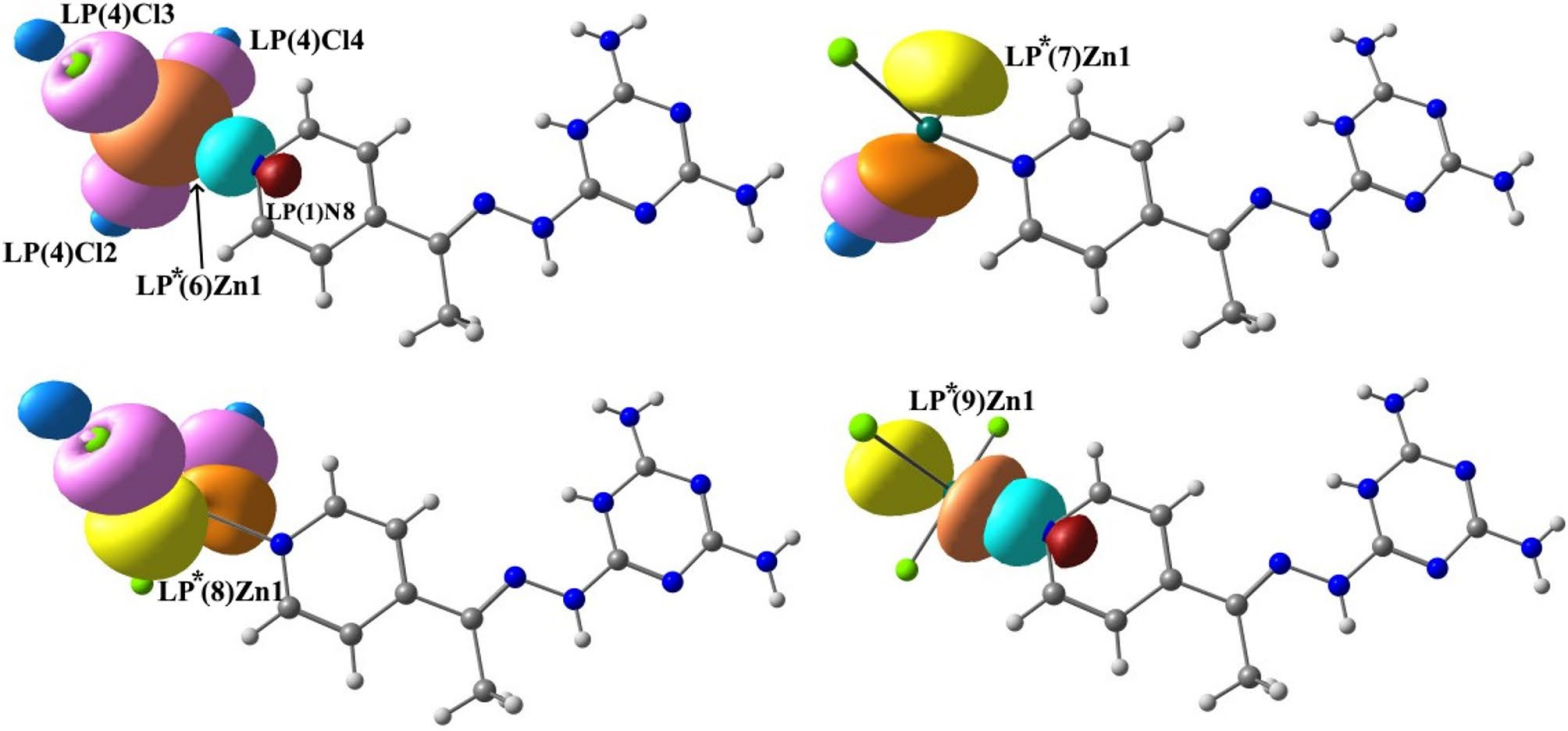
The coordination between the donor and acceptor NBOs in **1**.

Additionally, it is crucial to note that the LP*(6) orbital of Zn(II) serves as the most significant anti-bonding natural orbital, contributing to all Zn-N and Zn-Cl interactions as tabulated in Table S7 and shown in Fig. 12. Furthermore, the LP*(6) anti-bonding NBO is the most occupied anti-bonding orbital with 0.3871 e, compared to the other acceptor NBOs (Table S8). It is found that, LP*(6)Zn primarily has s-orbital characters, while LP*(7)Zn and LP*(8)Zn have mainly p-orbitals character while LP*(9)Zn displays mainly a p-orbital character with a very little contribution from d-orbitals. As a result of these donor-acceptor interactions, the electronic configuration of Zn1 in **1** is [core]4 S^(0.38)^3d^(9.98)^4p^(0.63)^5p^(0.01)^.

In complex **2**, it is clear that the interaction energies for the Ag-N (*s*-triazine) and Ag-O (nitrate anion) bonds are highest in the Ag4 unit, calculated to be 32.4 and 29.41 kcal/mol, respectively. The Ag3 unit follows closely with values of 32.39 and 28.08 kcal/mol. In contrast, the respective interaction energies are low in the Ag2 unit at 21.31 and 20.11 kcal/mol, and Ag1 unit has the lowest values of 16.62 and 13.88 kcal/mol. According to the literature, lower coordination numbers usually lead to stronger bonds for each ligand, since metal orbitals are concentrated in fewer M–L interactions, which minimizes mutual repulsion among ligands^53,54^. Important orbital-orbital interactions in the four Ag units are graphically presented in Figs. S16 and S17. Also, the LP*(6)Ag is the most occupied anti-bonding orbital compared to the other acceptor NBOs. Its occupancy values are 0.1916 e, 0.2075 e, 0.2217 e, and 0.2358 e for Ag1, Ag2, Ag3, and Ag4 unit, respectively (Table S8, Supplementary Data). Within the four units, LP*(6)Ag anti-bonding NBO has mainly s-orbital contributions while the LP*(7)Ag, LP*(8) and LP*(9) have p-orbitals contributions with some d-orbitals character. It is observed that Ag3 and Ag4 units have almost similar E^(2)^ values and orbital-orbital interactions (Fig. S17). This could be due to the similar coordination environment around silver atom in the two units.

### Cytotoxicity assay

The cytotoxicity of **L** and its complexes is evaluated *in vitro* against hepatocellular (HepG-2) and breast (MCF-7) carcinoma cell lines in addition to human lung fibroblast (WI-38) normal cell line. Their activities were investigated using the MTT colorimetric assay method. The IC_50_ value, defined as the concentration needed to reduce cancer cell growth by 50%^55^, indicates the level of cytotoxicity of the compound under investigation. The IC_50_ results are summarized in Table 4 (*µ*M) and Table S9 (*µ*g/mL), while further details are provided in Tables S10-S12. Complex **2** has demonstrated promising efficiency against both selected cancer cell lines. The IC_50_ value against the HepG-2 cell line is 9.96 ± 0.35 *µ*M, while for the MCF-7 cell line, it is 15.84 ± 0.46 *µ*M. These values are significantly lower than those of *cis-*platin, a standard cancer medications, which has IC_50_ values of 62.44 ± 1.99 *µ*M for HepG-2 and 63.43 ± 2.32 *µ*M for MCF-7^56^. This aligns with the literature indicating that many of silver metal complexes are more effective as anticancer agents for treating different types of cancer compared to *cis-*platin^22,23^. The enhanced potency of **2** can be attributed to the presence of four silver centers^21^. In comparison to the free ligand, complex **2** also demonstrates markedly lower IC_50_ values. The free ligand has IC_50_ values of 1243.92 ± 42.91 *µ*M and 1719.44 ± 59.61 *µ*M against HepG-2 and MCF-7 cells, respectively. Moreover, **1** demonstrates very high anticancer activity compared to the free ligand (**L**) against both cancer cell lines. Its IC_50_ values are 239.64 ± 12.77 *µ*M for HepG-2 and 281.82 ± 8.52 *µ*M for MCF-7. In conclusion, complex **2** displays superior potency against both cancer cell lines, being around 125, 24, and 6 times more effective than **L**, **1**, and *cis*-platin against HepG-2 cell, respectively, as well as, 108, 18, and 4 times more effective against MCF-7 cell, respectively.

Based on the comparison of **1** with previously reported Zn(II)-*s*-triazine complexes, it is evident that **1** exhibits greater activity than [Zn(BPT^Morph^)(H_2_O)_3_](ClO_4_)_2_^57^ complex, where, BPT^Morph^ = 4-(4,6-*bis*(3,5-dimethyl-1*H*-pyrazol-1-yl)-1,3,5-triazin-2-yl) morpholine, against the same two cancer cell lines. The latter has IC_50_ of 273.58 ± 10.24 and 310.74 ± 8.34 *µ*M against HepG-2 and MCF-7 cancer cell lines, respectively. Additionally, **1** demonstrates superior activity compared to [Zn(BPT^Morph^)(NO_3_)_2_]^57^ complex, which has an IC_50_ of 418.35 ± 10.59 *µ*M against the MCF cancer cell line. Furthermore, **2** outperformed the previously reported polymeric [Ag(HL2)(OClO_3_)]_n_·nH_2_O complex^58^, where, HL2 = 3-aminopyrazine-2-carboxylic acid, against MCF-7 cells, which showed an IC_50_ value of 130.48 ± 2.66 *µ*M, indicating eightfold greater cytotoxicity. Against HepG-2 cells, **2** showed slightly lower cytotoxicity compared to the [Ag(MTZ)_2_NO_3_] complex^59^(MTZ = 2-methyl-5-nitroimidazole-1-ethanol) which exhibited an IC_50_ of 8.03 ± 0.5 *µ*M after 72 h of incubation.

Additionally, the ligand and its two complexes demonstrated great antiproliferative activity on cancer cells as indicated by a selectivity index (SI) greater than 1 for all compounds, as shown in Table 4. As the SI values increase, the effect on cancerous cells becomes more pronounced while inflicting minimal damage to normal cells. The highest SI values are observed for complex **2**, with values of 4.1 against HepG-2 and 2.6 towards MCF-7 carcinoma cells. These results are slightly lower than those of *cis*-platin, which are 5.0 and 4.9, respectively (Table 4). The ability of zinc compounds to promote Lewis activation, nucleophile formation, and rapid ligand exchange underlies their catalytic activity in hydrolytic reactions, including DNA cleavage, which is relevant to antitumor effects^60,61^. However, the cytotoxicity mechanism of Ag(I) complexes primarily involves the release of intracellular Ag(I) ions in biological fluids, facilitated by weak Ag-N and Ag-O bonds^41^, which subsequently promote DNA and /or protein interaction^62–64^, apoptosis induction^65^, and enzyme inhibition^66^. Additionally, the biological study regarding the antimicrobial activity **L** and its complexes **1** and **2** has been evaluated.

**Table 4:** Cytotoxicity (IC_50_, *µ*M) of the three compounds studied and *cis*-platin^56^

Compound	HepG-2	SI	MCF-7	SI	WI-38
**L**	1243.92 ± 42.91	≥ 1.6	1719.44 ± 59.61	≥ 1.2	≥ 2047
**1**	239.64 ± 12.77	1.9	281.82 ± 8.52	1.6	458.80 ± 15.29
**2**	9.96 ± 0.35	4.1	15.84 ± 0.46	2.6	40.82 ± 0.89
*cis*-Platin	62.44 ± 1.99	5.0	63.43 ± 2.32	4.9	311.44 ± 10.36

### Antimicrobial assay

For *in vitro* antimicrobial activity, the synthesized ligand and its two complexes were tested against various pathogenic bacteria and fungi. The test was performed using the agar diffusion technique. The inhibition zone diameters and minimum inhibitory concentration (MIC) values of the investigated compounds are tabulated in Table 5. The results show that complex **2** exhibited a broad spectrum of antimicrobial activity against all tested microbial cell lines. **L** exhibited no activity against the Gram-positive bacteria; however, both complexes were effective against *B. subtilis*. Complex **2** has a promising inhibition zone diameter and MIC value of 27 mm and 3.71 *µ*M, respectively, exceeding that of gentamicin (positive control), while **1** recorded a respective value of 20 mm and 354.97 *µ*M. Additionally, complex **2** was the only one active against *S. aureus* exhibiting an inhibition zone diameter of 16 mm compared to 25 mm for gentamicin. The three compounds demonstrate activity against the two studied Gram-negative bacterial cell lines, with the exception of ligand **L**, which showed no effect on *E. coli*. Complex **2** exhibited superior efficiency compared to **L** and complex **1** against both studied Gram-negative bacteria. **2** inhibited *E. coli* and *P. vulgaris* at 22 and 13 mm, respectively. These results are very comparable to gentamicin, as indicated in Table 5. On the other hand, the **L** and complex **2** have moderate and similar activity against fungi, while **1** is found inactive. The inhibition zone diameters for **L** and complex **2** are 8 and 9 mm, respectively, against *A. fumigates*, while the diameter is 10 mm against *C. albicans* for both of them. The research revealed that the antibacterial efficacy of the metal complexes was primarily influenced by the metal ion, with zinc(II)^27^ and silver(I)^35^ complexes showing a stronger effect compared to their free ligands. In comparison, **1** exhibited higher antibacterial activity against *(B) subtilis* than ZnCl_2_^67^ (12 mm) and the other complexes such as [ZnL(ONO_2_)_2_]^27^; **3** and [ZnL(NCS)_2_]^27^; **4**, where **L** is 2,4-*bis*(morpholin-4-yl)-6-[(*E*)-2-[1-(pyridin-2-yl)ethylidene]hydrazin-1-yl] 1,3,5-triazine, which exhibited inhibition zones of 15 and 16 mm, respectively. Additionally, **3** and **4** were inactive against *E. coli*, while **1** showed an observable activity.

Comparing the antimicrobial activity of **2** with AgNO_3_, it is evident that **2** exhibited greater effectiveness against all microbial cells, except *P. vulgaris*. In this case, AgNO_3_ has an inhibition zone with a diameter of 20 mm (see Table 5). In contrast, both **2** and AgNO_3_ display a value of 10 mm against *C. albicans*. Further comparisons were made between **2** and the previously reported [Ag₂(2ClDAT)₂(NO₃)₂(H₂O)]^35^; **5** (2ClDAT = 2-chloro-4,6-diamino-1,3,5-triazine), and [Ag(BPAT)(ACN)]ClO_4_^50^; **6** complexes (Table 5). Complex **2** demonstrated superior activity against *B. subtilis* and *E. coli* when compared to **5** and **6**. Moreover, **2** was found to be more potent against *S. aureus* than **5**, and against *P. vulgaris* than **6.** Additionally, **2** was found to be more potent against *C. albicans* than **4**, whereas **3** was inactive. Additionally, **5** showed no antifungal activity towards the tested fungi, whereas **2** was active.

**Table 5:** Antimicrobial activities for **L**, **1** and **2**

Microorganism	L	ZnCl_2_^c^	AgNO_3_	1	2	3^d^	4^d^	5^e^	6^f^	Control
*Gram-positive bacteria*										
*S. aureus*	NA	16	14 (376.76)	NA	16 (120.70)	14	15	12	20	25 (30.31)^a^
*B. subtilis*	NA	12	13 (1839.64)	20 (354.97)	27 (3.71)	15	16	13	23	27(10.05)^a^
*Gram-negatvie bacteria*										
*E. coli*	NA	15	15 (188.38)	9 (˃ 5000)	22 (60.35)	NA	NA	9	16	30 (10.05)^a^
*P. vulgaris*	11 (˃ 5000)	–	20 (918.35)	11 (2840.65)	13 (482.96)	18	20	14	11	26 (10.05)^a^
*Fungi*										
*A. fumigates*	8 (˃ 5000)	–	NA	NA	9 (1931.83)	10	NA	NA	–	18 (293.92)^b^
*C. albicans*	10 (˃ 5000)	–	10 (753.52)	NA	10 (965.91)	9	10	NA	13	20 (588.03)^b^

Values for diameter of inhibition zone (mm) are outside the parentheses, and values for MIC (*µ*M) are inside. NA, not active; ^a^ Gentamicin; ^b^ Ketoconazole; ^c^ ref (^67^); ^d^ ref (^27^); ^e^ ref (^35^), and ^f^ ref (^50^).

Obviously, the antimicrobial activity of the complexes is strongly influenced by ligand design^68^, metal ion identity^68–70^ and coordination geometry^68,71^. *s*-Triazine based ligands are regarded as an important structural scaffold due to the presence of three nitrogen donor atoms, which result in electron delocalization and contribute to superior antimicrobial activity by increasing lipophilicity and cellular uptake^72^. Additionally, the enhanced antimicrobial activity of complex **2** compared to **1** can be rationalized based on chelation theory^73^. In **1**, the ligand coordinates in a monodentate manner, whereas in **2**, it acts as a multidentate chelator around the Ag(I), leading to the formation of a stable chelate ring. This chelation further increases the lipophilicity of **2** more than **1**, which facilitates Ag(I) complex penetration through biological membranes and inhibits various cellular enzymes that are crucial for the diverse metabolic pathways of microbes^74,75^. These observations highlight the critical role of ligand chelating capability as it reflects on the biological efficacy of the complexes as bacteriostatic agents compared to their free ligands^76^.

## Experimental

### Materials and methods

The supplementary data provides a detailed description of all chemicals and materials utilized in the synthesis, and also the characterization methods applied.

### Syntheses

#### Synthesis of the organic ligand (**L**)

The **L** ligand was prepared by applying the procedure mentioned in Method S1^77,78^ and outlined in Scheme 1. The characterization result of **L** using FT-IR is presented in Fig. S1, whereas the NMR spectra are displayed in Figs. S4 and S7.

**L** (white powder): Yield; C_10_H_12_N_8_; 75%; m.p. = 318–320 °C. Anal. Calc. C, 49.17; H, 4.95; N, 45.88. Found: C, 49.09; H, 4.98; N, 45.79. FT-IR (KBr, cm^− 1^): 3364, 3196 ν(N–H), 1584, 1540 ν(C = N), 1478 ν(C = C) (Fig. S1). ^1^H NMR (DMSO-*d*_*6*_): *δ* = 9.57 (s, H, -NH, exchangeable with D_2_O), 8.54 (d, 2 H, 2CH), 7.66 (d, 2 H, 2CH), 6.37 (s, 4 H, 2NH_2_, exchangeable with D_2_O), 2.21 (s, 3 H, CH_3_) ppm (Fig. S4). ^13^C NMR (DMSO-*d*_*6*_): *δ* = 167.9, 165.9, 150.2 ,146.4, 144.1 ,120.7, 13.3 ppm (Fig. S7).

#### Synthesis of complexes **1** and **2**

An aqueous solution of the metal salt, ZnCl_2_ (0.1 mmol, 13.63 mg) or AgNO_3_ (0.2 mmol, 33.97 mg) was added dropwise to a warm ethanolic solution of **L** (0.1 mmol 24.42 mg) (Scheme 1). The obtained solution after filtration was allowed to evaporate slowly. Complex **1** (pale yellow) and **2** (colorless) were formed after 5 and 12 days, respectively, in crystalline cubic shape.

[Zn(HL)Cl_3_] complex (**1**). Yield; C_11_H_16_Cl_3_N_8_O_0.5_Zn; 88%; m.p. = 276–278 °C. Anal. Calc. C, 30.02; H, 3.66; N, 25.46; Zn, 14.86. Found: C, 30.06; H, 3.72; N, 25.38; Zn, 14.75. FT-IR (KBr, cm^− 1^): 3517 ν (O–H), 3432, 3346 ν(N–H), 3167, 3090 ν(C-H), 1586, 1541 ν(C = N), 1491, 1463 ν(C = C), 1188 ν(C–N) (Fig. S2). ^1^H NMR (DMSO-*d*_*6*_): *δ* = 9.97 (broad s, 1H, NH, exchangeable with D_2_O), 8.55 (d, 2 H, 2CH), 7.77 (d, 2 H, 2CH), 6.56 (s, 4 H, 2NH_2_, exchangeable with D_2_O), 2.25 (s, 3 H, CH_3_) ppm (Fig. S5). ^13^C NMR (DMSO-*d*_*6*_): *δ* = 167.5, 165.4, 150.3 ,147.4, 121.7, 100.0, 13.5 ppm (Fig. S8).

[Ag_4_(HL)_2_(NO_3_)_6_]_n_ complex (**2**). Yield; C_20_H_26_Ag_4_N_22_O_18_; 70%; m.p. = 260–262 °C. Anal. Calc. C, 18.56; H, 2.03; N, 23.81; Ag, 33.34. Found: C, 18.49; H, 2.05; N, 23.72; Ag, 33.22. FT-IR (KBr, cm^− 1^): 3318, 3216 ν(N–H), 1628, 1593 ν(C = N), 1532 ν(C = C), 1377 ν(N-O), 1174 ν(C–N) (Fig. S3).^1^H NMR (DMSO-*d*_*6*_): *δ* = 10.74 (s, 1 H, -NH, exchangeable with D_2_O), 8.18 (s, 2 H, 2CH), 7.6 (s, 2 H, 2CH), 7.22 (s, 4 H, 2NH_2,_ exchangeable with D_2_O), 2.37 (s, 3 H, CH_3_) ppm (Fig. S6). ^13^C NMR (DMSO-*d*_*6*_): *δ* = 166.1, 163.6, 151.5 ,148.2, 122.8, 100.0, 16.5 ppm (Fig. S9).

### Crystal structure determination

Single crystal structures of both complexes were examined as mentioned in Method S2^79–82^. The crystal data and refinement parameters are tabulated in Table S1.

### Computational studies

The *CrystalExplorer 21.5* software^83^ was used to perform Hirshfeld analysis^84^. On the other hand, Gaussian 09 program was used for DFT calculations, selecting ωB97XD method and GENECP keyword, includeing LANL2DZ and 6-31G(d, p) basis sets for metals and non-metals, respectively, for both complexes^85,86^.

### Biological evaluation

Method S3 was used to assess the anticancer activity^87^, while Method S4 was used to test the antimicrobial activity^88^ of the organic ligand (**L**), as well as its Zn(II) and Ag(I) complexes. A comparison was made between the efficacy of these compounds to determine which one is more effective against different types of cancer cells and various microbial strains.

## Conclusion

This study describes the development and synthesis of two new complexes; the mononuclear [Zn(HL)Cl_3_] (**1**) and the polymeric tetranuclear [Ag_4_(HL)_2_(NO_3_)_6_]_n_ (**2**), utilizing a novel *s*-triazine- based ligand (**L**). In both complexes, the ligand is protonated (**HL**^**+**^) and is acting as a cationic ligand, which is uncommon in coordination chemistry. In **1**, the Zn-atom is tetra-coordinated, while **2** exhibits a combination of 3, 4, and 5 coordination numbers. Hirshfeld surface analysis revealed that the Cl···H (37.2%) and N···H (12.3%) interactions are critical for the crystal stability of **1** while **2** is predominantly stabilized by the O···H (50.4%) and O···Ag (7.8%) interactions. NBO analysis showed that LP*(6) orbital is the most occupied antibonding orbital in both complexes. As a result, the partial charges of the Zn(II) and Ag(I) are decreased than their formal values. Both complexes exhibit superior anti-cancer activity compared to the free ligand. Complex **2** demonstrates very strong effect against both cancerous cells, exceeding the performance of the anticancer drug *cis*-platin by 6 and 4 times versus HepG-2 and MCF-7 cells, respectively. The SI values were found to be greater than one for all compounds. These results motivate the* in vivo* applications and the design of a pharmaceutical based on this compound. Additionally, the most notable antimicrobial activity was observed for **2** towards *B. subtilis* with inhibition zone diameter and MIC outcomes of 27 mm and 3.71 *µ*M, respectively, matching that of ketoconazole as a positive control.

## Supplementary Information

Below is the link to the electronic supplementary material.

Supplementary Material 1

## Data Availability

All data generated or analyzed during this study are included in this manuscript.
