# Supplementary material for: Monomeric Zn(II) and 3D polymeric Ag(I) complexes with a cationic s-triazine hydrazone-based ligand; synthesis, structure, coordination behavior and biological studies
Source: Sci Rep. 2026 Aug 11;16:24915. doi: 10.1038/s41598-026-57114-z (PMC13462694; doi:10.1038/s41598-026-57114-z)
Supplement: Supplementary file 1 [file 41598_2026_57114_MOESM1_ESM.docx]

**Monomeric Zn(II) and 3D polymeric Ag(I) complexes with a cationic *s*-triazine hydrazone-based ligand; synthesis, structure, coordination behavior and biological studies**

**Eman M. Fathalla^1^*, Amal Yousri^1^, Ayman El-Faham^1,2^, Saied M. Soliman^1^, Assem Barakat^3^*, Roland C. Fischer^4^, Morsy A.M. Abu-Youssef^1^, Ahmed M. A. Badr^1^ & Sara M. Khattab^1^**

^1^Department of Chemistry, Faculty of Science, Alexandria University, P.O. Box 426, Ibrahimia, Alexandria 21321, Egypt

^2^Department of Basic Medical Sciences, College of Medicine, Dar Al Uloom University, P. O. Box 45142, Riyadh 11512, Saudi Arabia

^3^Department of Chemistry, College of Science, King Saud University, P.O. Box 2455. Riyadh 11451, Saudi Arabia

^4^Institute of Inorganic Chemistry, Graz University of Technology, Stremayrgasse 9/V, A-8010 Graz, Austria

*****Correspondence: [eman.nomeir@alexu.edu.eg](mailto:eman.nomeir@alexu.edu.eg), [ambarakat@ksu.edu.sa](mailto:ambarakat@ksu.edu.sa)

**Fig. S1.** FTIR of **L**.

**Fig. S2.** FTIR of complex **1**.

**Fig. S3.** FTIR of complex **2**.

**Fig. S4.** ^1^H NMR of **L** recorded in DMSO-*d_6_* (upper) and DMSO-*d_6_*/ D_2_O (lower).

**Fig. S5.** ^1^H NMR of complex **1** recorded in DMSO-*d_6_* (upper) and DMSO-*d_6_*/ D_2_O (lower).

**Fig. S6.** ^1^H NMR of complex **2** recorded in DMSO-*d_6_* (upper) and DMSO-*d_6_*/ D_2_O (lower).

**Fig. S7.** ^13^C NMR of **L**.

**Fig. S8.** ^13^C NMR of **1**.

**Fig. S9.** ^13^C NMR of **2**.

 **Fig. S10.** ORTEP diagram of **1** with thermal ellipsoids at 50% probability level.

**Fig. S11.** ORTEP diagram of **2** with thermal ellipsoids at 50% probability level.

**Fig. S12**. Hydrogen bond contacts: N-H···O (**A**), C-H···O (**B**), and their corresponding packing scheme (**C**) in complex **2**.

**Fig. S13**. The decomposed FP plots for all major and important contacts in [Zn(HL)Cl_3_] (**1**).

**Fig. S14**. The decomposed FP plots for all major and important contacts in [Ag_4_(HL)_2_(NO_3_)_6_]_n_ (**2**).

**Fig. S15**. The π···π stacking interaction appeared as red zones in the d_norm_ and green flat areas in the curvedness map of **2**. All contact distances are in angstroms.

**Fig. S16**. The NBOs’ interactions in Ag1 and Ag2 units of **2**.

**Fig. S17**. The NBOs’ interactions in Ag3 and Ag4 units of **2**.

**Table S1.** Crystal data for **1** and **2.**

|  | **1** | **2** |
| --- | --- | --- |
| CCDC | 2518341 | 2518342 |
| Empirical formula | C_11_H_16_Cl_3_N_8_O_0.5_Zn | C_20_H_26_Ag_4_N_22_O_18_ |
| Formula weight | 440.04 | 1294.11 |
| Temperature/K | 100.00(10) | 100.00(16) |
| Crystal system | monoclinic | triclinic |
| Space group | *C2/c* | *P-1* |
| *a*/Å | 13.1294(2) | 9.7218(2) |
| *b*/Å | 13.1797(2) | 13.6424(4) |
| *c*/Å | 19.7769(4) | 15.0910(4) |
| *α*/° | 90 | 72.241(3) |
| *β*/° | 102.1260(17) | 79.521(2) |
| *γ*/° | 90 | 69.464(2) |
| Volume/Å^3^ | 3345.86(10) | 1778.68(9) |
| Z | 8 | 2 |
| ρ_calc_g/cm^3^ | 1.747 | 2.416 |
| μ/mm^‑1^ | 6.586 | 18.429 |
| F(000) | 1784.0 | 1264.0 |
| Crystal size/mm^3^ | 0.27 × 0.05 × 0.04 | 0.11 × 0.08 × 0.06 |
| Radiation | Cu Kα (λ = 1.54184) | Cu Kα (λ = 1.54184) |
| 2Θ range for data collection/° | 9.148 to 155.016 | 6.172 to 155.68 |
| Index ranges | -16≤ h≤16, -16≤k≤16, -23≤l≤25 | -12≤h≤12, -17≤k≤17, -16≤l≤19 |
| Reflections collected | 28397 | 59560 |
| Independent reflections | 3534 [R_int_ = 0.0457, R_sigma_ = 0.0207] | 7499 [R_int_ = 0.0442, R_sigma_ = 0.0229] |
| Data/restraints/parameters | 3534/0/200 | 7499/0/579 |
| Goodness-of-fit on F^2^ | 1.145 | 1.058 |
| Final R indexes [I>=2σ (I)] | R_1_^a^ = 0.0363, wR_2_^b^ = 0.0913 | R_1_^a^ = 0.0279, wR_2_^b^ = 0.0630 |
| Final R indexes [all data] | R_1_^a^ = 0.0368, wR_2_^b^ = 0.0917 | R_1_^a^ = 0.0289, wR_2_^b^ = 0.0635 |
| Largest diff. peak/hole / e Å^-3^ | 0.43/-0.87 | 2.98/-0.94 |

*^a^* *R1* = Σ||*F*_o_| – |*F*_c_||/Σ|*F*_o_|. *^b^wR*_2_ = {Σ[*w*(*F*_o_^2^ – *F*_c_^2^)^2^]/ Σ[*w*(*F*_o_^2^)^2^]}^1/2^

**Table. S2**. Hydrogen bond specifications of **1** (in Å and°).

| **D-H...A** | **D-H** | **H…A** | **D…A** | **D-H…A** |
| --- | --- | --- | --- | --- |
| N3-H3···Cl3^i^ | 0.80 | 2.55 | 3.240(2) | 145 |
| N4-H4A···Cl2^ii^ | 0.88 | 2.45 | 3.180(2) | 140 |
| N4-H4B···N1^iii^ | 0.88 | 2.10 | 2.953(3) | 164 |
| N5-H5A···Cl3^i^ | 0.84 | 2.49 | 3.254(2) | 153 |
| N5-H5B···Cl2^iiii^ | 0.84 | 2.58 | 3.391(2) | 163 |
| C10-H10···Cl3^i^ | 0.95 | 2.63 | 3.534(3) | 160 |

Symm. code ^i^1-x,y,1/2-z; ^ii^1/2-x,1/2-y,-z*;* ^iii^1-x,y,-1/2-z*;* ^iiii^1/2+x,1/2-y,-1/2+z.

**Table S3.** Anion···π interactions in **1**.

| **Contact** | **Distance** | **Symm.code** | **Contact** | **Distance** | **Symm.code** |
| --- | --- | --- | --- | --- | --- |
| Cl3···N3 | 3.240 | 1-x,y,1/2-z | Cl3···C3 | 3.316 | x,1-y,-1/2+z |
| Cl3···N1 | 3.333 | x,1-y,-1/2+z | Cl3···C10 | 3.534 | 1-x,y,1/2-z |
| Cl3··· N5 | 3.254 | 1-x,y,1/2-z | Cl2···C6 | 3.529 | 1/2-x,1/2+y,1/2-z |
| Cl2··· N4 | 3.180 | 1/2-x,1/2-y,-z | Cl3···C1 | 3.409 | x,1-y,-1/2+z |
| Cl2··· N5 | 3.391 | -1/2+x,1/2-y,1/2+z | Cl2··· C4 | 3.556 | 1/2-x,-1/2+y,1/2-z |

**Table S4.** Hydrogen bonds for **2** (Å and º).

| **D-H**···**A** | **D-H** | **H**···**A** | **D**···**A** | **D-H**···**A** |
| --- | --- | --- | --- | --- |
| N4-H4A···O18^i^ | 0.88 | 2.14 | 2.875(4) | 141 |
| N5-H5A···O16 | 0.88 | 2.41 | 3.010(4) | 126 |
| N5-H5A···O13^ii^ | 0.88 | 2.58 | 3.002(4) | 111 |
| N8-H8···O10^iii^ | 0.88 | 1.98 | 2.774(4) | 150 |
| N13-H13A···O4^iv^ | 0.88 | 2.54 | 2.982(4) | 112 |
| N13-H13A···O6^iv^ | 0.88 | 2.22 | 3.072(4) | 162 |
| N16-H16···O17^v^ | 0.88 | 1.84 | 2.714(4) | 172 |
| C5-H5C···O15^vi^ | 0.98 | 2.45 | 3.348(4) | 152 |
| C7-H7···O11^i^ | 0.95 | 2.51 | 3.421(5) | 161 |
| C8-H8A···O12^iii^ | 0.95 | 2.34 | 3.110(4) | 137 |
| C15-H15A···O11^vii^ | 0.98 | 2.42 | 3.355(4) | 159 |
| C15-H15C···O3^viii^ | 0.98 | 2.58 | 3.447(5) | 148 |
| C17-H17···O6^ix^ | 0.95 | 2.42 | 3.198(5) | 139 |
| C18-H18···O5^ix^ | 0.95 | 2.49 | 3.266(5) | 139 |
| C19-H19···O16 | 0.95 | 2.55 | 3.320(5) | 138 |
| C20-H20···O1^x^ | 0.95 | 2.57 | 3.160(5) | 120 |
| C20-H20···O3^x^ | 0.95 | 2.58 | 3.515(4) | 169 |

Symm. code ^i^-1+x,y,z; ^ii^1-x,2-y,1-z; ^iii^1-x,1-y,2-z; ^iv^x,-1+y,1+z; ^v^1-x,2-y,-z;

^vi^-x,2-y,1-z; ^vii^1-x,1-y,1-z; ^viii^-x,1-y,1-z; ^ix^-x,2-y,-z; ^x^1+x,y,z.

**Table S5.** The π···π and anion···π stacking in **2**.

| **Contact** | **Distance** | **Symm.code** | **Contact** | **Distance** | **Symm.code** |
| --- | --- | --- | --- | --- | --- |
| **π ···π** | | | | | |
| C2···C9 | 3.397 | -x,2-y,1-z | N3···C6 | 3.413 | -x,2-y,1-z |
| C2···C10 | 3.240 | -x,2-y,1-z | N3···C10 | 3.375 | -x,2-y,1-z |
| C12···C20 | 3.211 | 1-x,1-y,1-z | N10···C20 | 3.403 | 1-x,1-y,1-z |
| C12···C19 | 3.300 | 1-x,1-y,1-z |  |  |  |
| **Anion···π** | | | | | |
| O5···C18 | 3.266 | -x,2-y,-z | O18···C18 | 3.234 | -x,2-y,-z |
| O6···C17 | 3.198 | -x,2-y,-z | O10···N8 | 2.774 | 1-x,1-y,2-z |
| O1···C20 | 3.160 | -1+x,y,z | O17···N16 | 2.714 | 1-x,2-y,-z |
| O1···C19 | 3.179 | -1+x,y,z | O5···N8 | 3.116 | -x,2-y,1-z |
| O12···C8 | 3.249 | -1+x,y,z |  |  |  |

**Table S6.** The E_xy_ for complexes **1** and **2**.

| **Contact** | **E_xy_** | **Contact** | **Exy** |
| --- | --- | --- | --- |
| **1** | | | |
| Zn···N | 2.4 | N···N | 1.3 |
| Zn···H | 1.3 | N···H | 0.9 |
| Cl···Cl | 0.1 | N···C | 1.8 |
| Cl···N | 0.6 | H···H | 0.9 |
| Cl···H | 1.5 | H···C | 0.6 |
| Cl···C | 1.0 | C···C | 2.2 |
| **2** | | | |
| N···N | 1.9 | C···Ag | 3.2 |
| N···C | 2.4 | O···O | 0.4 |
| N···O | 0.9 | O···H | 1.6 |
| N···H | 0.8 | O···Ag | 1.8 |
| N···Ag | 0.3 | H···H | 0.6 |
| C···C | 6.0 | H···Ag | 0.1 |
| C···O | 0.1 | Ag···Ag | 0.3 |
| C···H | 0.5 |  |  |

**Table S7.** Detailed orbital- orbital interactions of complexes **1** and **2**.

| **D** → **A NBOs** | **E^(2)^(kcal/mol)** | **D** → **A NBOs** | **E^(2)^(kcal/mol)** |
| --- | --- | --- | --- |
| **Complex 1** | | | |
| LP(1)Cl2 → LP*(7)Zn1 | 18.39 | LP(4)Cl3 → LP*(7)Zn1 | 28.71 |
| LP(2)Cl2 → LP*(8)Zn1 | 10.38 | LP(4)Cl3 → LP*(8)Zn1 | 58.19 |
| LP(3)Cl2 → LP*(9)Zn1 | 10.42 | LP(1)Cl4 → LP*(8)Zn1 | 12.11 |
| LP(4)Cl2 → LP*(6)Zn1 | 83.02 | LP(2)Cl4 → LP*(7)Zn1 | 7 |
| LP(4)Cl2 → LP*(7)Zn1 | 100.02 | LP(3)Cl4 → LP*(9)Zn1 | 8.68 |
| LP(1)Cl3 → LP*(7)Zn1 | 5.47 | LP(4)Cl4 → LP*(6)Zn1 | 77.64 |
| LP(1)Cl3 → LP*(8)Zn1 | 11.53 | LP(4)Cl4 → LP*(7)Zn1 | 23.95 |
| LP(2)Cl3 → LP*(7)Zn1 | 8.47 | LP(4)Cl4 → LP*(8)Zn1 | 69.99 |
| LP(3)Cl3 → LP*(9)Zn1 | 11.96 | LP(1)N8 → LP*(6)Zn1 | 32.22 |
| LP(4)Cl3 → LP*(6)Zn1 | 88.43 | LP(1)N8 → LP*(9)Zn1 | 49.75 |
| **Complex 2** | | | |
| **Ag1 unit** | | | |
| LP (1) N1 → LP*(6)Ag1 | 15.28 | LP (1) O14 → LP*(8)Ag1 | 8.44 |
| LP (1) N1 → LP*(7)Ag1 | 16.62 | LP (1) O14 → LP*(9)Ag1 | 8.58 |
| LP (1) N40 → LP*(8)Ag1 | 9.68 | LP (2) O14 → LP*(6)Ag1 | 9.82 |
| LP (1) N9 → LP*(6)Ag1 | 13.81 | LP (2) O14 → LP*(8)Ag1 | 6.42 |
| LP (1) N9 → LP*(7)Ag1 | 11.95 | LP (1) O3^#^ → LP*(6)Ag1 | 8.76 |
| LP (1) N9 → LP*(8)Ag1 | 5.1 | LP (1) O3^#^ → LP*(7)Ag1 | 9.09 |
| LP (1) N15 → LP*(6)Ag1 | 7.34 | LP (2) O3^#^ → LP*(6)Ag1 | 13.88 |
| LP (1) N15 → LP*(8)Ag1 | 7.55 | LP (2) O3^#^ → LP*(7)Ag1 | 6.04 |
| LP (1) N15 → LP*(9)Ag1 | 6.86 | LP (1) O69 → LP*(6)Ag1 | 5.25 |
| LP (1) O14 → LP*(6)Ag1 | 11.99 | LP (2) O69 → LP*(6)Ag1 | 6.26 |
| **Ag2 unit** | | | |
| LP (1) N2 → LP*(6)Ag2 | 21.31 | LP (2) O13^##^ → LP*(6)Ag2 | 10.85 |
| LP (1) N2 → LP*(8)Ag2 | 18.47 | LP (2) O13^##^ → LP*(7)Ag2 | 5.01 |
| LP (2) O3 → LP*(6)Ag2 | 5.79 | LP (1) O16^#^ → LP*(6)Ag2 | 5.5 |
| LP (2) O3 → LP*(7)Ag2 | 10.08 | LP (1) O16^#^ → LP*(7)Ag2 | 7.31 |
| LP (1) O1 → LP*(6)Ag2 | 5.2 | LP (2) O16^#^ → LP*(6)Ag2 | 20.11 |
| LP (1) O1 → LP*(8)Ag2 | 11.53 | LP (2) O16^#^ → LP*(7)Ag2 | 13.48 |
| **Ag3 unit** | | | |
| LP (1) N3 → LP*(6)Ag3 | 32.39 | LP (2) O4 → LP*(8)Ag3 | 5.44 |
| LP (1) N3 → LP*(8)Ag3 | 16.14 | LP (1) O17 → LP*(8)Ag3 | 7.38 |
| LP (1) O4 → LP*(8)Ag3 | 6.85 | LP (2) O17 → LP*(6)Ag3 | 20.61 |
| LP (2) O4 → LP*(6)Ag3 | 28.08 | LP (2) O17 → LP*(7)Ag3 | 6.42 |
| LP (2) O4 → LP*(7)Ag3 | 12.14 | LP (2) O17 → LP*(8)Ag3 | 7.76 |
| **Ag4 unit** | | | |
| LP (1) O7 → LP*(8)Ag4 | 6.61 | LP (1) O10 → LP*(8)Ag4 | 7.34 |
| LP (2) O7 → LP*(6)Ag4 | 25.35 | LP (2) O10 → LP*(6)Ag4 | 29.41 |
| LP (2) O7 → LP*(7)Ag4 | 10.27 | LP (2) O10 → LP*(7)Ag4 | 6.06 |
| LP (2) O7 → LP*(8)Ag4 | 6.17 | LP (2) O10 → LP*(8)Ag4 | 9.42 |
| LP (1) N10 → LP*(6)Ag4 | 32.42 | LP (2) O6 → LP*(7)Ag4 | 7.05 |
| LP (1) N1→ LP*(8)Ag4 | 17.95 |  |  |

Symmetry codes ^#^ -1+x, y, z; ^##^ -x,2-y,1-z.

**Table S8.** Occupancy of acceptor NBOs of the metal ions in complexes **1** and **2**.

| **Acceptor** | **Occupancy** | **Acceptor** | **Occupancy** |
| --- | --- | --- | --- |
| **Complex 1** | | | |
| LP*(6)Zn1 | 0.38709 | LP*(8)Zn1 | 0.22264 |
| LP*(7)Zn1 | 0.23275 | LP*(9)Zn1 | 0.16355 |
| **Complex 2** | | | |
| **Ag1 unit** | | | |
| LP*(6)Ag1 | 0.19160 | LP*(8)Ag1 | 0.09921 |
| LP*(7)Ag1 | 0.10306 | LP*(9)Ag1 | 0.07823 |
| **Ag2 unit** | | | |
| LP*(6)Ag2 | 0.20746 | LP*(8)Ag2 | 0.08238 |
| LP*(7)Ag2 | 0.09670 |  |  |
| **Ag3 unit** | | | |
| LP*(6)Ag3 | 0.22173 | LP*(8)Ag3 | 0.06750 |
| LP*(7)Ag3 | 0.08389 |  |  |
| **Ag4 unit** | | | |
| LP*(6)Ag4 | 0.23579 | LP*(8)Ag4 | 0.06943 |
| LP*(7)Ag4 | 0.08737 |  |  |

**Table S9**. Cytotoxicity (IC_50_, *µ*g/mL) of the three compounds studied and *cis*-platin.

| Compound | HepG-2 | SI | MCF-7 | SI | WI-38 |
| --- | --- | --- | --- | --- | --- |
| L | 303.84 ± 10.48 | ≥1.6 | 419.99± 14.56 | ≥1.2 | ≥ 500 |
| 1 | 105.45± 3.12 | 1.9 | 124.01± 3.75 | 1.6 | 201.89± 6.73 |
| 2 | 12.89± 0.45 | 4.1 | 20.50± 0.59 | 2.6 | 52.82± 1.15 |
| *cis*-Platin | 18.8 ± 0.6 | 5.0 | 19.1 ± 0.7 | 4.9 | 93.45±3.11 |

**Table S10.** Evaluation of the cytotoxicity of **L** against tested cell lines.

| **Cancer cell line** | **HepG-2** | | | **MCF-7** | | | **WI-38** | | |
| --- | --- | --- | --- | --- | --- | --- | --- | --- | --- |
| conc. (*µ*M) | Viability % | Inhibitory % | S.D. (±) | Viability % | Inhibitory % | S.D. (±) | Viability % | Inhibitory % | S.D. (±) |
| 2046.999 | 27.81 | 72.19 | 2.73 | 36.28 | 63.72 | 3.13 | 54.23 | 45.77 | 3.11 |
| 1023.500 | 56.09 | 43.91 | 1.82 | 79.15 | 20.85 | 2.07 | 86.48 | 13.52 | 2.65 |
| 511.750 | 81.42 | 18.58 | 0.74 | 93.26 | 6.74 | 0.72 | 91.56 | 8.44 | 0.62 |
| 255.875 | 94.57 | 5.43 | 0.45 | 99.41 | 0.59 | 0.36 | 98.79 | 1.21 | 0.75 |
| 127.937 | 99.03 | 0.97 | 0.12 | 100 | 0 |  | 100 | 0 |  |
| 63.866 | 100 | 0 |  | 100 | 0 |  | 100 | 0 |  |
| 31.933 | 100 | 0 |  | 100 | 0 |  | 100 | 0 |  |
| 15.967 | 100 | 0 |  | 100 | 0 |  | 100 | 0 |  |
| 0.000 | 100 | 0 |  | 100 | 0 |  | 100 | 0 |  |

**Table S11.** Evaluation of the cytotoxicity of complex **1** against tested cell lines.

| **Cancer cell line** | **HepG-2** | | | **MCF-7** | | | **WI-38** | | |
| --- | --- | --- | --- | --- | --- | --- | --- | --- | --- |
| conc. (*µ*M) | Viability % | Inhibitory % | S.D. (±) | Viability % | Inhibitory % | S.D. (±) | Viability % | Inhibitory % | S.D. (±) |
| 1136.260 | 7.46 | 92.54 | 0.32 | 10.23 | 89.77 | 1.09 | 16.28 | 83.72 | 2.14 |
| 568.130 | 19.57 | 80.43 | 1.45 | 27.69 | 72.31 | 2.15 | 40.89 | 59.11 | 1.73 |
| 284.065 | 43.21 | 56.79 | 2.13 | 49.58 | 50.42 | 3.42 | 64.56 | 35.44 | 2.19 |
| 142.033 | 64.92 | 35.08 | 1.84 | 76.05 | 23.95 | 1.76 | 83.44 | 16.56 | 1.02 |
| 71.016 | 79.56 | 20.44 | 1.02 | 88.41 | 11.59 | 0.73 | 93.57 | 6.43 | 0.71 |
| 35.451 | 90.43 | 9.57 | 0.65 | 96.25 | 3.75 | 0.24 | 99.02 | 0.98 | 0.54 |
| 17.726 | 98.56 | 1.44 | 0.17 | 99.67 | 0.33 | 0.11 | 100 | 0 |  |
| 8.863 | 100 | 0 |  | 100 | 0 |  | 100 | 0 |  |
| 0.000 | 100 | 0 |  | 100 | 0 |  | 100 | 0 |  |

**Table S12.** Evaluation of the cytotoxicity of complex **2** against tested cell lines.

| **Cancer cell line** | **HepG-2** | | | **MCF-7** | | | **WI-38** | | |
| --- | --- | --- | --- | --- | --- | --- | --- | --- | --- |
| conc. (*µ*M) | Viability % | Inhibitory % | S.D. (±) | Viability % | Inhibitory % | S.D. (±) | Viability % | Inhibitory % | S.D. (±) |
| 386.366 | 1.04 | 98.96 | 0.26 | 2.19 | 97.81 | 0.56 | 4.02 | 95.98 | 0.46 |
| 193.183 | 3.75 | 96.25 | 0.31 | 6.43 | 93.57 | 0.31 | 11.28 | 88.72 | 1.03 |
| 96.591 | 9.63 | 90.37 | 0.59 | 15.84 | 84.16 | 1.24 | 27.59 | 72.41 | 1.77 |
| 48.296 | 17.98 | 82.02 | 1.44 | 26.02 | 73.98 | 0.79 | 43.21 | 56.79 | 2.15 |
| 24.148 | 29.47 | 70.53 | 1.39 | 41.56 | 58.44 | 2.43 | 65.14 | 34.86 | 2.72 |
| 12.055 | 45.18 | 54.82 | 2.16 | 53.87 | 46.13 | 1.65 | 79.45 | 20.55 | 0.91 |
| 6.027 | 59.03 | 40.97 | 1.85 | 68.91 | 31.09 | 0.75 | 92.34 | 7.66 | 0.52 |
| 3.014 | 75.29 | 24.71 | 0.76 | 82.35 | 17.65 | 0.43 | 97.13 | 2.87 | 0.29 |
| 0.000 | 100 | 0 |  | 100 | 0 |  | 100 | 0 |  |

**Materials and Physical Measurements**

All chemicals and solvents were purchased from Sigma-Aldrich Chemical Company Inc. (St. Louis, MO, USA) and were utilized without additional purification. The FTIR spectra were obtained using a Bruker Tensor 37 FTIR instrument, Bruker, Germany, employing the KBr disc method and recording the spectra in the range of 4000–400 cm^-1^. ^1^H NMR and ^13^C NMR spectra were measured using JEOL JNM-ECZR spectrometer (500 MHz) in CDCl_3_. A PerkinElmer 2400 Elemental Analyzer was employed for CHN analysis (PerkinElmer Inc., Waltham, MA, USA). The metal content was determined using a Shimadzu atomic absorption spectrophotometer (AA7000 series, Shimadzu, Ltd., Kyoto, Japan).

**Method S1**. Synthesis of the organic ligand **(L)**.

The (*E*)-6-(2-(1-(pyridin-4-yl)ethylidene)hydrazineyl)-1,3,5-triazine-2,4-diamine (**L**) was prepared as follow: The first step was achieved to substitute two chlorine atoms of cyanuric chloride using the previously mentioned method^77^. Cyanuric chloride (922 mg, 5 mmol) dissolved in 7.5 mL of acetone was added to 7.5 mL of ice water, creating a fine suspension. A solution of NH_4_OH (10 mL, 1 N, 9.9 mmol) was then added gradually to the suspension while maintaining a temperature of 0°C. The resulting mixture was stirred for 30 minutes at 0 °C and then for an additional 30 minutes at 40 °C. The product was formed as white solid, which was then filtered, washed with water (3 x 5ml), and dried under vacuum. It was subsequently purified by column chromatography using ethylacetate/ hexane (2:1) as the eluent, resulting in highly pure 6-chloro-1,3,5- triazine-2,4-diamine. The second step involves replacing the last chlorine by the hydrazine, which was carried out using a reported method^78^. (2.5 g, 17.18 mmol) of 6-chloro-1,3,5- triazine-2,4-diamine was suspended in 20 mL of water, then hydrazine hydrate (4.32 g, 4.2 mL, 86.4 mmol) was added dropwise. The mixture was refluxed overnight at 85˚C then cooled at room temperature. The white solid was separated by filtration, washed with distilled water (400 mL) then dried to under high vacuum. The pure 2,4-diamino-6- hydrazinyl-1,3,5-triazine is obtained (1.80 g, 72%). The new ligand (**L**) was synthesized by dissolving 0.71 g (5 mmol) of the compound obtained in the second step in 25 mL of methanol, then 4-acetylpyridine (0.61 g, 0.56 mL, 5 mmol) was added dropwise during stirring, allow to stirring at rt overnight. The reaction was monitored by TLC until the end, filtration, washing with methanol (2x 50 mL), and finally dried under vacuum to obtain the new ligand with high purity.

**Method S2: X-Ray structure determinations**^79-82^.

The crystals of the two complexes was immersed in cryo-oil, mounted in a loop, and measured at a temperature of 100 K. The X-ray diffraction data were collected on a Rigaku XtaLAB Synergy, Dualflex, HyPix-Arc 100 diffractometer. The crystal was kept at 100.00(16) K with an Oxford Cryostream 800 cooling system during data collection with Cu Kα radiation. The *CrysAlisPro*^79^ software package was used for cell refinements and data reductions. A gaussian absorption correction (*CrysAlisPro*^79^) was applied to the intensities before the structure solutions. The structures were solved by the intrinsic phasing (*SHELXT*^80^) method. Structural refinements were carried out using *SHELXL*^81^ software with *Olex2*^82^ graphical user interface.

**Method S3. Evaluation of cytotoxicity activity^87^**

**Mammalian cell lines: WI-38** cells (human lung fibroblast normal cell line), hepatocellular (HepG-2), and breast (MCF-7) carcinoma cell lines were obtained from the American Type Culture Collection (ATCC, Rockville, MD).

**Chemicals Used:** Dimethyl sulfoxide (DMSO), MTT and trypan blue dye were purchased from Sigma (St. Louis, Mo., USA).

Fetal Bovine serum, DMEM, HEPES buffer solution, L-glutamine, gentamycin and 0.25% Trypsin-EDTA were purchased from Lonza (Belgium).

**Cell line Propagation:**

The cells were propagated in Dulbecco’s modified Eagle’s medium (DMEM) supplemented with 10% heat-inactivated fetal bovine serum, 1% L-glutamine, HEPES buffer and 50*µ*g/mL gentamycin. All cells were maintained at 37ºC in a humidified atmosphere with 5% CO_2_ and were subcultured two times a week.

**Cytotoxicity evaluation using viability assay:** For cytotoxicity assay, the cells were seeded in 96-well plate at a cell concentration of 1×10^4^ cells per well in 100*µ*L of growth medium. Fresh medium containing different concentrations of the test sample was added after 24 h of seeding. Serial two-fold dilutions of the tested chemical compound were added to confluent cell monolayers dispensed into 96-well, flat-bottomed microtiter plates (Falcon, NJ, USA) using a multichannel pipette. The microtiter plates were incubated at 37ºC in a humidified incubator with 5% CO_2_ for a period of 24 h. Three wells were used for each concentration of the test sample. Control cells were incubated without test sample and with or without DMSO. The little percentage of DMSO present in the wells (maximal 0.1%) was found not to affect the experiment. After incubation of the cells viable cells yield was determined by a colorimetric method.

After incubating for 24 hr, the numbers of viable cells were determined by the MTT test. Briefly, the media was removed from the 96 well plate and replaced with 100 *µ*L of fresh culture RPMI 1640 medium without phenol red then 10 *µ*L of the 12 mM MTT stock solution (5 mg of MTT in 1 mL of PBS) to each well including the untreated controls. The 96-well plates were then incubated at 37°C and 5% CO_2_ for 4 hr. An 85 *µ*L aliquot of the media was removed from the wells, and 50 *µ*L of DMSO was added to each well and mixed thoroughly with the pipette and incubated at 37°C for 10 min. Then, the optical density was measured at 590 nm with the microplate reader (SunRise, TECAN, Inc, USA) to determine the number of viable cells and the percentage of viability was calculated as [(ODt/ODc)]x100% where ODt is the mean optical density of wells treated with the tested sample and ODc is the mean optical density of untreated cells.

The relation between surviving cells and drug concentration is plotted to get the survival curve of each tumor cell line after treatment with the specified compound. The 50% inhibitory concentration (IC_50_), the concentration required to cause toxic effects in 50% of intact cells, was estimated from graphic plots of the dose response curve for each conc. using Graphpad Prism software (San Diego, CA. USA).

**Method S4: Antimicrobial studies^88^**

**a) Tested pathogenic microbes**

The antibacterial activity of the studied ligand and its complexes were evaluated against two Gram positive bacteria (*S. aureus (*ATCC 25923) and *B. subtilis* (RCMB015(1)NRR LB-543), two Gram negatvie bacteria ((*E. coli* (ATCC 25922) and *P. vulgaris* (RCMB 004(1)ATCC 13315) and two fungi (*A. niger* (RCMB 002008) and *C. albicans* (RCMB 005003(1) ATCC 10231). Gentamicin was used as a standard antibacterial agent. The samples maintained in Brain heart infusion (BHI) at 20^o^C; 300 mL of each stock–culture was added to 3 mL of BHI broth. Overnight cultures were kept for 24 hr at 37 ± 1^o^C and the purity of cultures was checked after 24 hr of incubation. After 24 hr of incubation, bacterial suspension was diluted with sterile physiological solution, for the diffusion and indirect bioautographic tests, to 108 CFU/mL (turbidity = McFarland barium sulfate standard 0.5). In case of fungi *A. niger* (RCMB 002008) and *C. albicans* (RCMB 005003(1) ATCC 10231), the used medium in antagonistic activity against tested fungi is Potato Dextrose Agar, where Fluconazole was used standard antifungal agent.

b) **Agar well diffusion method**

Different metal complexes were prepared at concentration 10 mg/mL dissolved in DMSO as stock solutions. Preparation of sterilized Mueller Hinton agar plates seeded with tested pathogenic bacteria occurred. The wells are done by sterilized cork borer in size 6 mm and hence 200 *µ*g of the synthetic compound was poured in each well comparably with DMSO as control. The plates were incubated at 37°C for 24 hr. after incubation period; antimicrobial activity was determined by inhibition zones.

**References**

77. Das, P. & Mandal, S. K. Understanding the effect of an amino group on the selective and ultrafast detection of TNP in water using fluorescent organic probes. *J. Mater. Chem. C* **6**, 3288–3297 (2018).

78.	Zhang, Q., He, C., Yin, P. & Shreeve, J. M. Insensitive Nitrogen-Rich Materials Incorporating the Nitroguanidyl Functionality. *Chem. - An Asian J.* **9**, 212–217 (2014).

79. Rigaku OD. *CrysAlis PRO* (Rigaku Oxford Diffraction, 2015).

80.	Sheldrick, G. M. SHELXT–Integrated space-group and crystal-structure determination. *Found. Crystallogr.* **71**, 3–8 (2015).

81. Sheldrick, G. M. Crystal structure refinement with SHELXL. *Cryst. Struct. Commun.* **71**, 3–8 (2015).

82.	Hübschle, C. B., Sheldrick, G. M. & Dittrich, B. ShelXle: a Qt graphical user interface for SHELXL. *Appl. Crystallogr.* **44**, 1281–1284 (2011).

87. Mosmann, T. Rapid colorimetric assay for cellular growth and survival: application to proliferation and cytotoxicity assays. *J. Immunol. Methods* **65**, 55–63 (1983).

88.	Wayne, P. A. Clinical and laboratory standards institute. Performance standards for antimicrobial susceptibility testing (2011).
